# Defining and predicting outcomes of a work-injuries rehabilitation program through multivariate baseline phenotypes and longitudinal modeling

**DOI:** 10.1371/journal.pone.0362083

**Published:** 2026-11-04

**Authors:** Mohammad Bayattork, Tom Carter, David M. Walton

**Affiliations:** 1 School of Physical Therapy, Western University, London, Ontario, Canada; 2 Department of Sport Sciences and Physical Education, Faculty of Humanities Science, University of Hormozgan, Bandar Abbas, Iran; 3 CBI Health Group Inc., Toronto, Ontario, Canada; Versiti Blood Research Institute, UNITED STATES OF AMERICA

## Abstract

**Objective:**

To characterize short-term pain and health-related quality-of-life (HRQoL) recovery trajectories in injured workers undergoing standardized musculoskeletal rehabilitation. We also examined whether multidimensional baseline phenotypes derived from brief self-report measures predicted these trajectories and subsequent recovery.

**Methods:**

Adults with work-related musculoskeletal injuries were enrolled at entry into a community-based rehabilitation program (N = 146), of whom 134 contributed to pain and 138 to HRQoL trajectory analyses. Participants were classified into one of 4 latent profiles at intake from a prior analysis: Localized/Resilient (57.0%), Mood-Dominant (21.8%), Neurosensory-Dominant (12.7%), and Complex/Central (8.5%). Pain (Numeric Pain Rating Scale, NPRS) and HRQoL (EQ-5D-5L index) were measured at baseline, 4, and 8 weeks; self-rated global recovery, self-reported return-to-work status, and administrative discharge/ongoing-care codes were collected at 8 weeks. Growth mixture modeling defined NPRS and EQ-5D-5L trajectories. Odds ratios (ORs) quantified associations between baseline classes and “No Recovery/No Improvement” trajectories, as well as point outcomes.

**Results:**

Three pain trajectories (Rapid Recovery 34.1%, Incomplete Recovery 42.2%, No Recovery 23.7%) and two QoL trajectories (Good QoL 86.2%, No Improvement 13.8%) were identified. Compared with the Localized/Resilient baseline class, odds of NPRS No Recovery were higher in the Mood-Dominant (OR 3.97, 95%CI 1.32–11.92), Neurosensory‑Dominant (OR 10.99, 3.20–37.38), and Complex, Central (OR 22.67, 4.76–107.90) classes, with similarly graded increases for EQ‑5D‑5L No Recovery (ORs 9.00, 7.50, and 150.00, respectively), although estimates for the small Complex/Central subgroup were imprecise. The Complex /Central class also had higher odds of self-reported “not fully returned to work” (OR 4.18, 1.05–16.64) and being coded as “requires ongoing care” (OR 5.74, 1.93–17.11) relative to Localized/Resilient, whereas self-rated global recovery showed no significant between-class differences.

**Conclusions:**

Brief, fully self-reported, multivariate phenotypes obtained at program entry stratify acutely injured workers into likely rehabilitation outcomes in the short term. These findings support the potential value of integrating mechanism-informed phenotyping with trajectory modeling to inform stratified, risk-guided musculoskeletal rehabilitation.

## Introduction

Musculoskeletal (MSK) conditions are among the leading causes of disability worldwide, accounting for a substantial proportion of years lived with disability and placing a major burden on health systems, individuals, and employers [1]. In Canada, MSK pain and injury represent a major contributor to work-related disability, with chronic pain alone generating substantial indirect costs through reduced workforce participation and increased healthcare utilization [2]. Accordingly, work-related and trauma-related MSK injuries remain a key concern in both public health and occupational rehabilitation. Improving recovery and return to work continues to be an important focus for clinicians and policymakers [3,4].

Contemporary rehabilitation for work-related musculoskeletal injuries generally emphasizes early, active, and individualized care, incorporating education and self-management, progressive exercise and graded activity, and strategies to support functional restoration and return to work [5]. In Ontario, these principles are operationalized through the Workplace Safety and Insurance Board (WSIB) Musculoskeletal Program of Care, an eight-week community-based rehabilitation pathway based on evidence-informed principles. Although the program provides a standardized framework for care, the selection, frequency, and intensity of specific interventions are individualized according to clinical findings, functional needs, and return-to-work goals [6].

Recovery following non-catastrophic MSK trauma is highly heterogeneous, with some people demonstrating rapid improvement while others experience delayed or minimal recovery of pain and function [7]. Traditional analytic approaches that summarize outcomes at a few time points risk obscuring this heterogeneity and may mask distinct subgroups whose trajectories differ meaningfully in level and rate of change [8]. To address this, growth mixture modeling (GMM) and related latent class approaches have increasingly been used to identify unobserved subgroups with distinct longitudinal patterns of pain or function after trauma [8–10]. Prior work has identified two to five clinically interpretable trajectories of recovery, dependent on the outcome, population, and sample size [7,11,12]. Across studies, commonly observed patterns include a favorable or rapid recovery trajectory and a persistent or minimal recovery trajectory, with one or more intermediate, delayed, or fluctuating patterns identified in many cohorts [11]. Such trajectory-based approaches provide greater richness on the pathways patients travel rather than just the single point outcome at follow-up.

There is growing recognition that recovery from MSK conditions is not a singular, uniform variable but rather a complex, multidimensional process that unfolds over time [13,14]. It can include non-linear improvements in pain, physical function, participation in work, and broader impacts on daily life that may occur at different rates. Importantly, the definition of recovery itself is inconsistent. For some, recovery may mean a reduction or resolution of symptoms, while for others it may be more closely tied to returning to meaningful roles, activities, or a sense of normalcy in everyday life [4,7,15]. These insights have led to greater use of multiple complementary recovery indicators, including self-reported global recovery, work participation, and administrative outcomes such as health resource utilization. Importantly, brief patient-reported measures have demonstrated utility in capturing these broader recovery dimensions in routine clinical settings [16].

There is limited evidence on how multivariate baseline profiles, combining symptom severity, psychological distress, and related clinical features, map onto early pain and quality of life trajectories over the course of funded work injury-specific rehabilitation [17–19]. Recent work has begun to address this gap by deriving prognostic phenotypes from multidimensional intake data and examining their relationships with subsequent recovery patterns [20,21]. As part of this research program, baseline patient-reported data from injured workers were used to derive multivariate phenotypes that represent distinct patterns across several pain-related domains [cite when available]. The present study examines whether membership in these baseline phenotypes is associated with subsequent recovery trajectories and indicators of case escalation. Prior analyses from this program of research showed that baseline prognostic indicators in acutely injured workers had variable relationships with recovery across social identity groups, with the single-scale TIDS and EQ-5D-5L demonstrating stronger prognostic performance than pain intensity alone [22]. In a subsequent study we used baseline (intake) self-report data from acutely injured workers to identify four latent classes (clusters) that appeared to hold clinical meaning in this sample [cite when available] and a simpler three-class solution that appeared robust to patient population (i.e., acutely injured workers and military veterans with chronic pain). One of the proposed uses of the multivariate classes is as a triaging tool for identifying risk of poor outcome after rehabilitation and potentially for directing workers to the most targeted treatment pathway.

In this longitudinal cohort study of adults receiving outpatient rehabilitation after musculoskeletal trauma within a large Canadian community provider, we first sought to characterize the recovery trajectories from a standardized 8-week rehabilitation program, then to estimate the prognostic accuracy of the multivariate classes for discriminating those who report good and poor rehabilitation outcomes. Based on prior evidence, we hypothesized two to four statistically and clinically meaningful latent trajectories within the longitudinal pain and HRQoL data, with at least two representing overall favorable and less favorable outcomes. We further hypothesized that baseline multivariable phenotype membership would be able to discriminate between longitudinal trajectories with at least 75% classification accuracy.

## Methodology

### Study design

This was a prospective longitudinal cohort study that is part of a larger project (clinicaltrials.gov ID NCT06016647) conducted in collaboration with a network of community-based physiotherapy clinics providing Workplace Safety and Insurance Board (WSIB)-funded protocol-driven care in Ontario, Canada. It is reported according to the STROBE (Strengthening the Reporting of Observational Studies in Epidemiology) statement [23]. It was conducted in accordance with principles of ethical research and received approval from the Western University Research Ethics Board (HSREB) prior to initiating recruitment (protocol #121883). All participants provided electronic (digital) consent prior to providing any data.

### Participants

Data were collected between October 2023 and May 2025 from outpatient physiotherapy clinics in Ontario, Canada that deliver care under the WSIB Musculoskeletal Program of Care (MSK PoC). The MSK PoC is an evidence-informed, community-based rehabilitation pathway of up to eight weeks, organized into two four-week blocks of care. Block one includes the initial assessment and a minimum of five treatment sessions, while block two includes a minimum of four additional sessions; treatment frequency and duration within these requirements are determined by the treating clinician according to the worker’s clinical presentation, functional recovery needs, and return-to-work goals. Recommended components include education and pain/self-management strategies, activity modification, individualized and progressive exercise therapy, manual therapy, and adjunct therapeutic modalities where clinically indicated. Education is a required component and is integrated with strategies to promote activity, functional recovery, and safe return to work. Specific treatment combinations were selected by the treating physiotherapist according to clinical judgement rather than prescribed by the research team.

Participants were adults (≥18 years) receiving treatment for non-catastrophic, work-related MSK injuries and able to complete questionnaires in English. Exclusion criteria were those who reported cognitive or comprehension difficulties that would interfere with completing the questionnaires, as well as those with conditions requiring substantially different levels of care (e.g., active cancer, systemic infection, or neuromuscular disorders).

Participants were recruited using a convenience sampling approach with voluntary opt-in participation. Eligible workers could enroll either by responding to an email invitation sent after their initial clinic visit or by accessing a study link displayed on posters in clinic waiting areas. Interested workers reviewed the study information and provided informed consent via a secure online platform (Qualtrics®, Seattle, WA, USA) before completing the survey. Participation was entirely voluntary and had no impact on clinical care or WSIB funding decisions.

### Measures

All data were self-reported directly by participants, save for one outcome extracted from an administrative database. The data used to assign participants to latent profile analysis (LPA)-based classes at entry into the MSK PoC have been described in detail elsewhere, including the process of creating the profiles [cite when available]. In brief, the questionnaires were drawn from a seven-axis framework of ‘pain drivers’, tapping each of the following: nociceptive (pain extent via body diagram), neuropathic (brief version of the Leeds Assessment of Neuropathic Signs and Symptoms, SLANSS [24]), central nociplastic (central symptoms subscore of the MultiDimensional Symptom Index, MSI_central_ [25]), beliefs about pain (brief Pain Catastrophizing Scale, briefPCS [26]), negative affect (Patient Health Questionnaire-4, PHQ4 [27]), sensorimotor incongruence (Fremantle Body Awareness Questionnaire, FreBAQ_general_ [28]), and experiences of discrimination due to visible intersectional identity (Intersectional Discrimination Index – Major, InDI-M [29]). Prior maximum likelihood-based latent profile analysis using Z-transformed scores assigned each participant to their most likely class based on posterior probabilities. In this sample of injured workers, and as reported previously [cite when available], the final profile solution indicated 4 meaningful phenotypes based on intake data: 55.0% were classed as ‘Localized/Resilient’ representing a group that scored well below the sample mean (better) across all indicator variables at the start of the MSK PoC. An additional 19.9% were classed as “Mood-Dominant” representing a group that scored above the mean on the psychologically-oriented variables (PHQ4, briefPCS), 14.7% were classed as “Neurosensory-Dominant” representing a group that scored above the mean on the neurologically-oriented variables (briefSLANSS, FreBAQ_general_), and 10.5% were classed “Central/Complex” representing a group that scored well above the mean (worse) across all indicators. Those classes are fully described in [cite when available].

Follow-up occurred at the midpoint (4 weeks) and end (8 weeks) of the initial MSK PoC (Fig 1). Participants indicated whether they had sustained a new injury or other health condition that changed their status within the MSK PoC between successive follow-up periods, with those indicating a new injury removed from the trajectory analysis to prevent contamination of the models. Recovery outcomes were collected in five different ways, two of which were repeated and are referred to as ‘trajectory outcomes’, while three were collected only at program completion and are referred to as ‘point outcomes’. The *trajectory outcomes* were the Numeric Pain Rating Scale (NPRS) assessed at its worst, and best over the past 24 hours and pain right now, with the variable of interest being the mean of all three. The second trajectory variable was the EuroQoL five-dimension, five-level (EQ-5D-5L [30],) quality of life metric for which scores were converted into index values from 0.00 (worst possible health) to 1.00 (best possible health) according to Canadian normative data. Both of the trajectory variables were collected at all three data collection periods.

**Fig 1:**
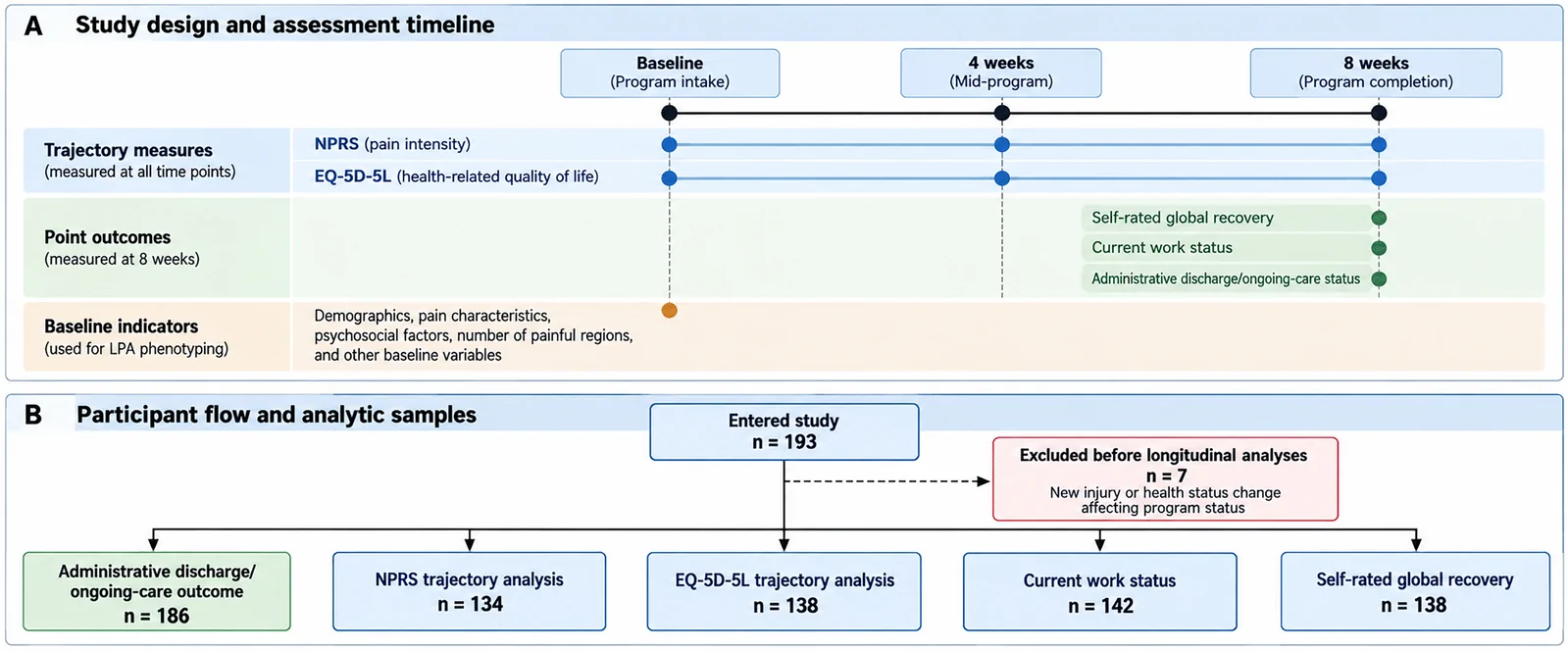
Study design, assessment timeline (A), and participant flow (B).

Three *point outcomes* were also collected at the end of the treatment period (8 weeks) only. Two were reported directly by the participant: General recovery status (Fully recovered, Mostly but not fully recovered, Not close to recovered) and work status in relation to pre-injury (Fully returned to work, Partially returned, or Not yet returned). The final point outcome was extracted from the CBI Health clinical database using a proprietary coding system to indicate whether participants were considered recovered by the clinician and/or funder at the time (recovered, no further care required) or required additional PoC or alternate levels of care (not recovered). Upon completion of the second (8-week) follow-up, study participation was complete (Fig 1).

### Analysis

**Data Fidelity and Cleaning**. From the full database, we first identified only those respondents who completed a minimum of two out of the three collection periods, and who did not indicate a new injury suffered at any point during the study. Missing values were then imputed using expectation maximization (EM) imputation assuming a random pattern of missing values (Little’s p > 0.05). We did not attempt to impute any missing point outcomes, rather participants were removed analysis-by-analysis if those were missing.

**Descriptives**. Participant demographics (age, sex, education, income) and baseline data were collated and interpreted descriptively (means and ranges, medians, or frequencies as appropriate).

**Point Outcomes**. Recovery status at 2-month follow-up was dichotomized as ‘Recovered’ or ‘Not Recovered’ for each participant using the three different recovery indicators. Self-rated recovery (‘Mostly or Fully Recovered’), self-rated work status (‘Fully back to pre-injury work hours and duties’), or administrative indicator based on clinical data (Discharged from rehabilitation care and back to work) were all coded as ‘Recovered’ and any other selections were considered ‘Not Recovered’ for these analyses.

**Recovery Trajectories.** Growth Mixture Modeling (GMM) was used to identify the distinct recovery trajectories across the three data collection periods for the NPRS and EQ-5D-5L. For base analyses all parameters were free to vary, only constrained as minimally necessary to achieve model convergence, and then only as long as the resulting model made conceptual and clinical sense. Constraints were applied in the order of residuals over time within each class, intercepts across classes, and finally slopes across classes and only if required. Goodness of fit was evaluated across several parameters: the model with the lowest Aikake (AIC) and sample-size adjusted Bayesian (adj.BIC) Information Criteria, highest entropy (>0.80), average posterior probabilities (APP) of class assignment >80% within each class, and no class containing <10% of the sample were used as omnibus fit indicators. An additional inferential criterion was the Lo-Mendell-Rubin adjusted Likelihood Ratio Test (LMR adj.LRT) that compares the fit of the k to k-1 class structures, for which a significant value (p < 0.05) indicates stronger fit of the k-class model. Finally, clinical meaningfulness was evaluated through visual and qualitative exploration of the trajectories. Trajectory classes were not defined using *a priori* clinical thresholds. Following selection of the optimal GMM solution, descriptive class labels were assigned post hoc based on the estimated mean level and pattern of change across the three assessment occasions. Each respondent was then assigned to one of the trajectories based on the highest posterior probability; the descriptive labels did not influence class assignment. The GMM procedures were conducted in MPlus v8.11 (© Muthen & Muthen).

**Prediction of Recovery Trajectories**. With each participant assigned to one baseline class from the prior LPA, and one of the newly derived trajectory classes, cross-tabulation matrices were constructed from which odds ratios, and 95% confidence intervals were calculated. For each analysis, the referent categories (LPA and outcomes) were coded with the goal of identifying the magnitude of increased odds of poor recovery, calculated for each LPA class as a referent against each other class. OR was considered significant if the lower 95% confidence limit did not include 1.00. As we anticipated very small sample sizes in some cells, these are presented as estimates of anticipated effect sizes only, useful for sample size calculations in future studies.

**Sample size**. No formal *a priori* sample-size calculation was performed. Sample-size requirements for growth mixture modeling are not determined by a single conventional threshold and depend on factors including class separation, relative class size, model complexity, and parameter specification. Previous trajectory studies in this field have used samples ranging from approximately 100–300 participants, including our prior work in acute musculoskeletal injury identifying two to three recovery trajectories [7]. Accordingly, the trajectory models and class proportions were interpreted as sample-dependent estimates requiring replication in larger cohorts.

## Results

### Data fidelity and cleaning

A total of n = 193 unique participants entered the study (Fig 1), of which seven reported a new injury over the course of the study and were removed. From the CBI Health administrative database, we were able to recover discharge status for all n = 186 who did not report a new injury while in the study. For the self-reported outcomes, n = 146 (78.5% of those with no new injuries) provided at least partial data for the longitudinal analyses. N = 11 (7.6%) were missing two NPRS data points, and n = 7 (4.8%) were missing two EQ-5D-5L and were removed for those respective analyses. There were no differences between those who provided complete data and those who did not in terms of age, sex, or NPRS score at entry (all p > 0.60). EM-based imputation was required for 37 total NPRS scores (9.2% of all included scores) and 23 total EQ-5D-5L scores (5.6%). Little’s test indicated the patterns of missing data were random (NPRS *χ*^2^ = 7.52, p = 0.28; EQ-5D-5L *χ*^2^ = 4.04, p = 0.67). This left n = 134 for the NPRS analyses and 138 for the EQ-5D-5L analyses. For the point outcomes at two months, the sample sizes were: Current work status (n = 142), Self-rated Global Recovery (n = 138), and Administrative discharge status (n = 186) (Fig 1).

### Descriptives

Participant descriptives are reported in Table 1. The sample was 64.1% female, with a mean age of 43.4 years (range 20–73); 44.4% had a university degree, and 55.4% earned over CAD$60,000/year. The clinical variables on intake indicated a moderately affected group (NPRS mean = 4.6/10; EQ-5D-5L mean = 0.62/1.00). Table 1 also presents the proportion of multivariate class assignments within the final sample as derived from the prior LPA.

**Table 1:** Participant characteristics.

Variable	Full sample (n = 146)
Sex (% female)	64.10%
Age (mean, range)	43.4 (20 to 73)
Educational Attainment	
≤high school	19.30%
Community or Technical college	36.30%
University	44.40%
Annual Household Income (% >CAD$60,000)	55.40%
Injury Region	
Head and neck	24.80%
Trunk	39.30%
Upper extremity	51.70%
Lower extremity	49.70%
NPRS /10 (mean, range)	4.6 (0.0 to 10.0)
TIDS /24 (mean, range)	10.1 (0.0 to 24.0)
EQ-5D-5L /1.0 (mean, range)	0.62 (0.00 to 0.95)
Multivariate Baseline Classes (% of sample)	
Localized/Resilient	57.00%
Mood-Dominant	21.80%
Neurosensory-Dominant	12.70%
Complex, Central	8.50%
PoC Point Outcomes (after 8 weeks)	
Fully or Mostly Recovered (self-rated)	21.70%
Fully Returned to Work (self-rated)	47.90%
Discharged from Rehabilitation (admin)	54.70%

NPRS = Numeric Pain Rating Scale (mean of 3 ratings); TIDS = Traumatic Injuries Distress Scale; EQ-5D-5L = EuroQoL 5-dimension, 5-level version; PoC = Program of Care.

### Point outcomes

Point outcomes and associations with sociodemographic variables have been reported in a prior paper [22] and are summarized here briefly. The proportion of participants classed as ‘recovered’ differed across the different recovery definitions used. Based on the self-reported global recovery question, 21.7% indicated they were mostly or fully recovered at 8 weeks, 47.9% reported they had returned to full pre-injury work duties, and 54.7% were coded as fully back to work and not requiring ongoing rehabilitation care after completion of the initial MSK PoC in the CBI Health administrative database.

### Derivation of longitudinal NPRS and EQ-5D-5L trajectories

For the NPRS data, attempting to extract either a 2- or a 4-class model led to inadmissible parameter estimates with non-positive definite residual covariance matrices suggesting model misspecification (2-class) or over-specification (4-class). Accordingly, the statistics are not trustworthy and are not reported. The 3-class unconstrained model converged normally, replicated across random starts, and appeared to yield a clinically meaningful trajectory structure. For the 3-class model: AIC = 1561.0, adj.BIC = 1557.3; Entropy = 0.88; APPs = 94.2% to 97.1%; LMR-adj.LRT = 18.1 (p < 0.01). Fig 2 graphically depicts the mean (95%CI) NPRS values for each class at each time. Based on the curve shapes, the classes were named: 1. Rapid Recovery (34.1% of the sample); 2. Incomplete Recovery (42.2%); 3. No Recovery (23.7%). Table 2 compares the mean values at each time point for each class. Rapid Recovery was characterized by a significant improvement of an average 1.6 NPRS points across each time point (p < 0.01 each). Incomplete Recovery showed a significant improvement of 0.9 points over the first 4 weeks (p < 0.01) but no further improvement from weeks 4–8 (p = 0.15). Mean NPRS scores in the No Recovery group showed no significant change across the full 8-week period (all p ≥ 0.10).

**Table 2:** Mean NPRS scores (/10) for each of the 3 classes across each of the data collection periods.

	Baseline	1-month	2-month
Class 1: Rapid Recovery (34.1%)	3.7 (3.1, 4.3)^1^	1.7 (1.3, 2.1)^2^	0.6 (0.4, 0.8)^3^
Class 2: Incomplete Recovery (42.2%)	4.5 (4.0, 5.0)^1^	3.6 (3.2, 3.9)^2^	3.3 (3.1, 3.5)^2^
Class 3: No Recovery (23.7%)	6.0 (5.4, 6.7)^1^	6.1 (5.7, 6.6)^1^	6.6 (6.3, 6.8)^1^

Superscript numbers represent adjacent values that are significantly different per repeated measures analysis of variance with Bonferroni correction (all p < 0.01), where different numbers (1, 2, 3) indicate significant differences between adjacent cells within a row, while the same numbers (1, 1, 1) indicate that the mean values those two cells are not significantly different within that row. Comparisons violated Mauchly’s Test of Sphericity, meaning a Greenhouse-Geiser correction was applied to the ANOVA results. NPRS = Numeric Pain Rating Scale.

**Fig 2:**
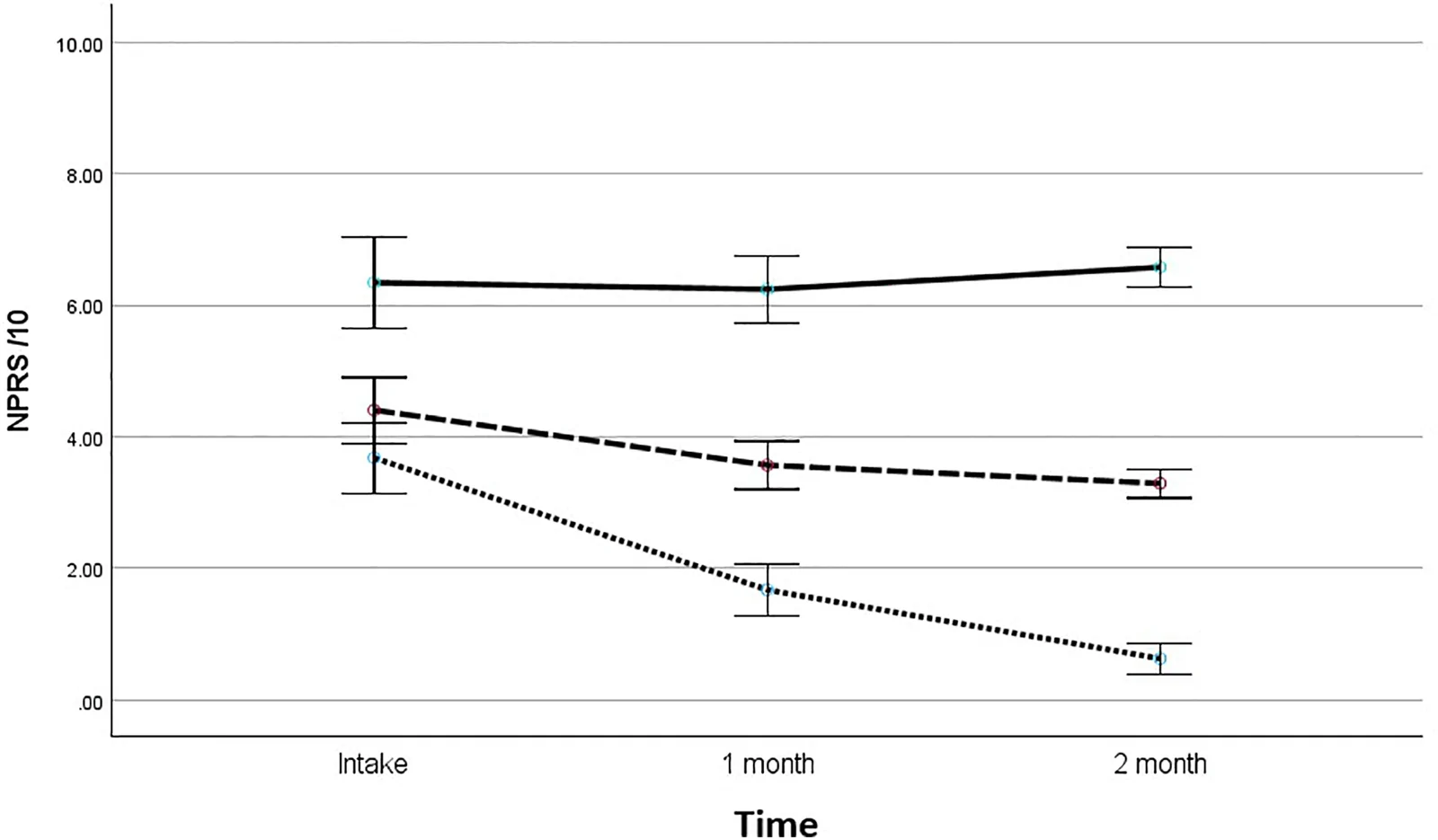
Trajectories of pain intensity across time by recovery class.

The EQ-5D-5L data demonstrated greater model sensitivity to specification choices. An unconstrained 3-class model converged though it produced a small class (6.5% of the sample) whose membership and size were unstable when slope variance constraints were imposed, effectively eliminating statistical support for a meaningful third class. A four-class unconstrained model also converged but included two classes with ≤6.5% of the sample, violating our acceptability criteria. A two-class model provided the most stable and interpretable representation of the data, but only when residual variances were constrained as equal across time within each trajectory class (Two-class model: AIC = −434.9, adj.BIC = −437.0; Entropy = 0.93; APP = 96.5% to 98.3%; LMR-adj.LRT = 49.87 (p = 0.01)). This constraint improved model stability but imposed equal residual variance across the three assessment occasions, thereby limiting the model’s ability to represent changes in within-class heterogeneity over the course of rehabilitation. Full model fit statistics for EQ-5D-5L trajectories are reported in S1 Table. Fig 3 graphically depicts the mean EQ-5D-5L values for each class at each time. The two-class model yielded two clinically interpretable trajectories: a large “Good Outcome” trajectory class (86.2% of the sample) with a mean baseline EQ-5D-5L score of 0.67 (95% CI 0.63, 0.70) improving at a significant mean rate of 0.07 points/month to reach 0.81 (95% CI 0.79, 0.83) after 8 weeks. A smaller “No Recovery” trajectory class (13.8%) entered with a lower mean EQ-5D-5L score of 0.38 (95% CI 0.29, 0.47) at baseline and showed no significant change over time with a mean score of 0.34 (95% CI 0.26, 0.41) after 8 weeks. Table 3 presents comparisons of mean values within each trajectory Fig 3.

**Table 3:** Mean EQ-5D-5L scores (/1.0) for the 2 classes across each of the data collection periods.

	Baseline	1-month	2-month
Class 1: Good Outcome (86.2%)	0.67 (0.63, 0.70)^1^	0.75 (0.72, 0.78)^2^	0.81 (0.79, 0.83)^3^
Class 2: No Recovery (13.8%)	0.38 (0.29, 0.47)^1^	0.33 (0.27, 0.39)^1^	0.34 (0.29, 0.40)^1^

Superscript numbers represent adjacent values that are significantly different per repeated measures analysis of variance with Bonferroni correction (all p < 0.01), where different numbers (1, 2, 3) indicate significant differences between adjacent cells within a row, while the same numbers (1, 1, 1) indicate that the mean values those two cells are not significantly different within that row. Comparisons violated Mauchly’s Test of Sphericity, meaning a Greenhouse-Geiser correction was applied to the ANOVA results. EQ-5D-5L = EuroQol 5-Dimension 5-Level.

**Fig 3:**
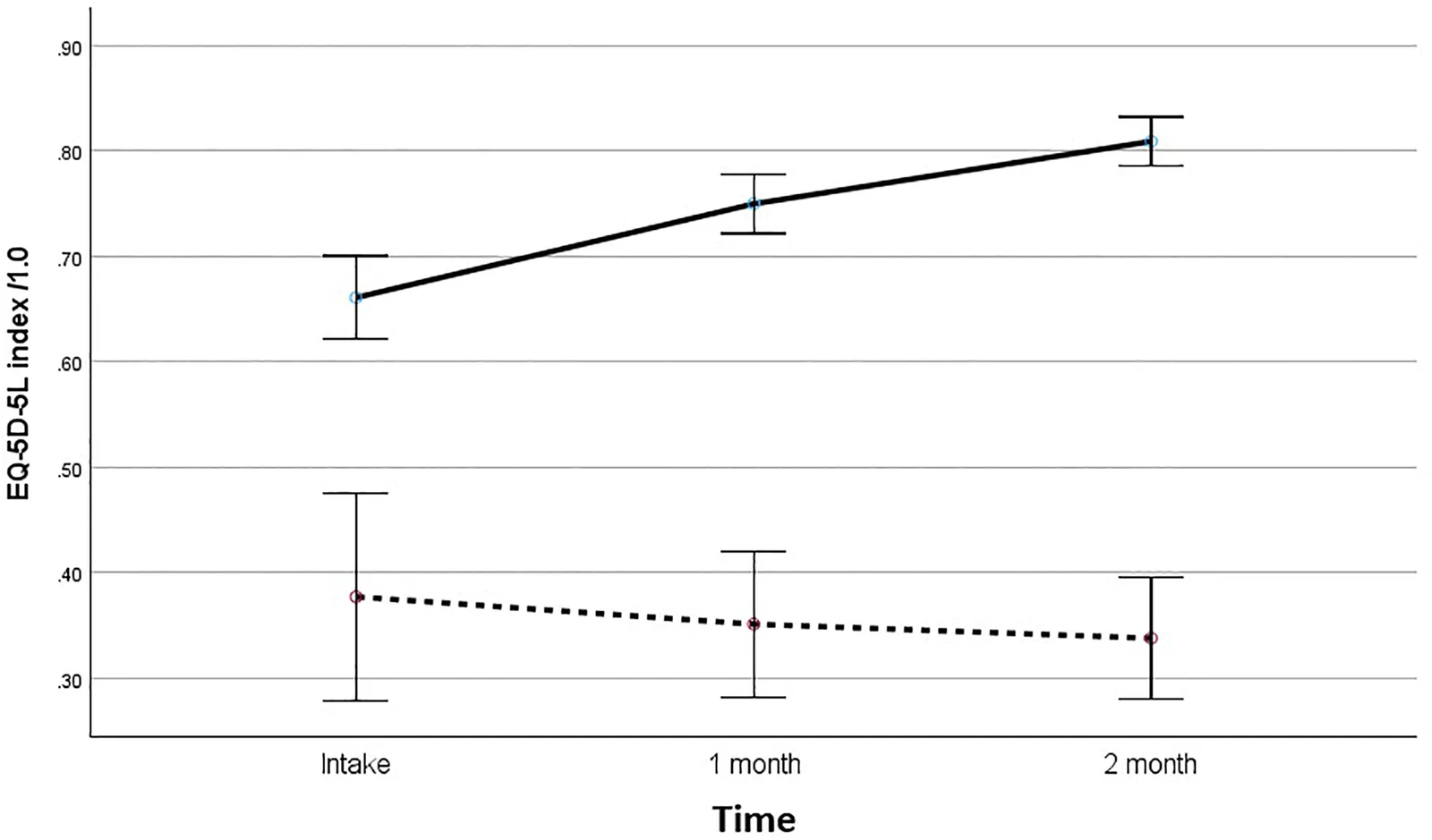
Trajectories of quality of life across time by recovery class.

### Prediction of recovery trajectories

Across all recovery trajectories and point outcomes, n = 133–146 had enough baseline and outcomes data to enable meaningful comparison of predictive accuracy when participants were classified according to intake profile and outcome group. Table 4 presents the proportions of participants within each 8-week outcome category by baseline LPA class, and Table 5 presents the odds ratios (OR, 95%CI) of each head-to-head comparison of LPA classes by each of the five outcome variables. Using the Localized/Resilient LPA class and the worst (no recovery) outcome category as the reference categories, the Complex/Central class showed significantly higher odds of poor outcome in four of the five comparisons (ORs = 4.18 to 150.00; Table 5). The exception was when self-rated recovery was the outcome being predicted, for which no LPA class showed significantly higher odds than any other for classifying two-month recovery status. The OR for non-recovery status was also significantly higher for the Mood-Dominant and Neurosensory-Dominant LPA classes compared to the Localized/Resilient class when NPRS or QoL trajectories were the outcome. A similar pattern was seen when self-rated work status was the outcome. For none of the comparisons were the odds of non-recovery significantly different between the Mood-Dominant and Neurosensory-Dominant LPA classes.

**Table 4:** Proportions (percent) of participants within each class based on intake data against the different longitudinal (NPRS, EQ-5D-5L) and point (self-rated recovery, self-rated work status, administrative discharge data) outcomes.

Recovery Trajectories or Indicators(2 months)	LPA Classes Based on Intake Data			
	Localized/Resilient(n = 82; 57%)	Mood-Dominant(n = 32; 22%)	Neurosensory-Dominant(n = 19; 13%)	Complex/Central(n = 13; 9%)
**A. NPRS trajectories**				
Rapid Recovery	45.3%	22.6%	11.8%	10.0%
Incomplete Recovery	45.3%	48.4%	35.3%	20.0%
No Recovery	9.3%	29.0%	52.9%	70.0%
**B. EQ-5D-5L trajectories**				
Good QoL	97.4%	80.6%	83.3%	20.0%
No Improvement	2.6%	19.4%	16.7%	80.0%
**C. Point outcomes at 8 weeks**				
Fully or Mostly Recovered (self-rated)	28.6%	16.1%	11.1%	0.0%
Fully Returned to Work(self-rated)	58.2%	36.7%	35.3%	25.0%
Discharged from Care(administrative)	58.8%	60.0%	61.1%	36.4%
				
**Percentage within baseline LPA class**				
0%	25%	50%	75%	100%

Superscript numbers represent chi-square comparisons of proportions across rows (Outcome) for each level of column (Baseline class), where cells with the same number are not significantly different (p > 0.05) while those with different numbers show different proportions (p < 0.05).

**Table 5:** Cross-tabulation of odds ratios, indicating odds of being classed in the ‘No Recovery’ group based on NPRS trajectories, with reference categories by row and comparator category by column.

*Referent Class*	*Odds Ratio (95% CI) of* *NPRS ‘No Recovery’ Trajectory*		
Mood-Dominant	Neurosensory-Dominant	Complex, Central
Localized/Resilient	**3.97 (1.32, 11.92)**	**10.99 (3.20, 37.38)**	**22.67 (4.76, 107.90)**
Mood-Dominant		2.75 (0.81, 9.39)	**5.73 (1.20, 27.12)**
Neurosensory-Dominant			2.07 (0.40, 10.85)
	*Odds Ratio (95%CI) of* ***QoL ‘No Recovery’ Trajectory***		
	Mood-Dominant	Neurosensory-Dominant	Complex, Central
Localized/Resilient	**9.00 (1.71, 47.48)**	**7.50 (1.15, 48.82)**	**150.00 (18.53, 1214.16)**
Mood-Dominant		0.83 (0.18, 3.84)	**16.67 (2.79, 99.57)**
Neurosensory-Dominant			**20.00 (2.75, 145.49)**
	*Odds Ratio (95%CI) of* ***Self-Rated ‘No Recovery’*** *response*		
	Mood-Dominant	Neurosensory-Dominant	Complex, Central
Localized/Resilient	2.08 (0.71, 6.11)	3.20 (0.68, 15.09)	8.00 (0.45, 142.95)
Mood-Dominant		1.54 (0.27, 8.89)	3.85 (0.19, 77.04)
Neurosensory-Dominant			2.50 (0.10, 61.22)
	*Odds Ratio (95%CI) of* ***Self-Rated ‘Not Returned to Work’*** *response*		
	Mood-Dominant	Neurosensory-Dominant	Complex, Central
Localized/Resilient	**2.41 (1.01, 5.73)**	**2.56 (0.86, 7.61)**	**4.18 (1.05, 16.64)**
Mood-Dominant		1.06 (0.31, 3.67)	1.74 (0.39, 7.81)
Neurosensory-Dominant			1.64 (0.32, 8.45)
	*Odds Ratio (95%CI) of* ***‘Requires Ongoing Care’ Coding*** *at end of PoC*^*1*^		
	Mood-Dominant	Neurosensory-Dominant	Complex, Central
Localized/Resilient	**2.25 (1.05, 4.84)**	1.78 (0.75, 4.19)	**5.74 (1.93, 17.11)**
Mood-Dominant		0.79 (0.29, 2.13)	2.55 (0.77, 8.48)
Neurosensory-Dominant			3.23 (0.91, 11.42)

**BOLD** = OR significantly > 1.00

1: Total N for the Requires Ongoing Care coding status was n = 186. For all other analyses, n = 133–137. OR = odds ratio; CI = confidence interval; NPRS = Numeric Pain Rating Scale; QoL = quality of life; PoC = Program of Care.

## Discussion

This study, the third in a series of analyses conducted using data from acutely injured workers, explored longitudinal data over the course of an 8-week rehabilitation program to identify potentially meaningful response trajectories and the prognostic utility of multivariate LPA classes. We have shown that multidimensional baseline phenotypes might be useful for estimating treatment outcomes whether those are defined as trajectories or single-point-in-time outcomes. In the two companion manuscripts we have reported on analyses that suggest the MSK PoC outcomes could be predicted by single scales (e.g., TIDS, EQ-5D-5L) or even a single item (NPRS) collected at entry, though with evidence of differential functioning across sociodemographic identities and limited direction for treatment decisions [22]. As a follow-up analysis, the seven-axis pain drivers framework informed the collection of multiple pain-related measures from which LPA identified four meaningful classes in the injured workers data. The multivariate LPA classes were proposed as more useful for identifying priority intervention targets, but their predictive value was unknown. This third study closes that loop.

In addition to the point outcomes (self-rated recovery, self-rated work status, and administrative end-of-PoC status), this study also identified latent trajectories within the early longitudinal pain intensity and quality of life data from injured workers. We identified three main NPRS trajectories: Rapid Recovery, Incomplete Recovery, and No Recovery; and two EQ-5D-5L trajectories: Good QoL and No Improvement. Those workers classed in the Localized/Resilient group, characterized by lower (better) mean scores across all intake variables at PoC entry, were consistently more likely to experience a positive outcome after the 8 weeks, with the only exception being when self-rated recovery was the outcome of interest for which no class was a better predictor. Conversely, those with more complex response patterns at entry, suggesting a dominant central or non-somatic presentation, were consistently more likely to experience a poor outcome after 8 weeks. The middle two classes (Mood-Dominant, neurosensory-dominant) showed inconsistent patterns of recovery. That all baseline data for LPA classification is based entirely on patient self-reported responses to standardized questionnaires, taking about five minutes to complete, offers clinical efficiency as a triage/risk screening tool, and the multivariate nature appears to provide clinicians with direction for further clinical workup where necessary.

In this context, our approach is conceptually and broadly comparable to well-known screening tools such as the Örebro Musculoskeletal Pain Screening Questionnaire (OMPQ) [31] and the STarT Back Screening Tool [32], which also use brief self-report questions to categorize MSK patients into risk groups for ongoing pain, disability, or work absence. In primary care groups with low back pain, STarT Back medium-risk categories generally have about 2–3 times higher odds of ongoing disabling back pain or poor functional results compared to low-risk groups, while high-risk categories have roughly 3–8 times higher odds over 6–12 months [33]. Additionally, STarT Back high-risk classifications have been linked to significantly higher odds of poor health-related quality of life and decreased work capacity compared with low-risk categories in acute or subacute back and neck pain [34]. Regarding work disability outcomes, findings using the ÖMPQ indicate that medium-risk scores relate to roughly double the risk of subsequent registered work disability, while high-risk scores are linked to approximately 10–16 times higher odds of long-term work disability or disability pension compared with low-risk scores over about two years [35]. The present findings should not be directly compared with those of STarT Back or the Örebro questionnaire because of differences in case mix, outcome definitions, and, importantly, follow-up duration. Whereas these established screening tools have commonly been evaluated over 6–12 months or longer, the present study evaluated outcomes only through completion of the 8-week MSK PoC and therefore addresses short-term prognostic associations. Nevertheless, the graded associations observed between baseline phenotypes and early recovery provide a rationale for examining whether these phenotypes retain prognostic value beyond program completion. Future prospective studies should evaluate whether baseline phenotype membership predicts sustained pain and HRQoL outcomes, durable return to work, and ongoing healthcare use at 6 and 12 months, and whether it provides prognostic information beyond established screening approaches.

The finding of three trajectories defined by a relevant clinical outcome (NPRS) aligns with prior GMM studies on acute and subacute MSK trauma [7,36]. About a third of our sample showed a pattern of rapid NPRS recovery and could be confidently labeled fully or near fully recovered after the MSK PoC across most of the outcome indicators. The remaining two-thirds, however, reported some degree of persistent symptoms across outcome indicators after the PoC and may represent a group for which standardized protocol-driven care could be supplemented with more targeted intervention based on baseline profile in the interest of optimizing treatment outcomes. The exceptions in our study were the EQ-5D-5L trajectories in which 86% showed satisfactory recovery and reported a mean index score improving from 0.67 to 0.81 (95% CI 0.79 to 0.83) after 8 weeks, ending just below a recently reported Canadian population mean of 0.86 [37]. All participants classified in the EQ-5D-5L ‘No Recovery’ trajectory also reported that they were far from recovery when directly queried. They also showed complex patterns of neurosensory, psychological, and central symptoms at intake, suggesting that signs of case escalation are identifiable early in the rehabilitation process. While eligibility for entering the MSK PoC required that the dominant symptoms could be attributed to injuries of the MSK system and precluded people with strong cognitive, emotional or more severe injuries, our findings may indicate a subgroup with complex health conditions that are not well-suited to the MSK PoC. This appears to be a promising direction for future research focused on improving system efficiencies and outcomes for all workers.

Results of the NPRS and EQ-5D-5L trajectories deserve some caution in interpretation. First, the sample available for mixture modeling was relatively modest, particularly for identifying and estimating smaller latent classes. The prognostic comparisons involving the Complex/Central phenotype should also be interpreted cautiously because this was the smallest baseline subgroup. Several odds ratios therefore had wide confidence intervals, indicating substantial statistical imprecision. This was particularly evident for the association between the Complex/Central phenotype and the EQ-5D-5L No Recovery trajectory (OR = 150.00, 95% CI 18.53–1214.16). Accordingly, the very large point estimate should not be interpreted literally as a precise estimate of effect magnitude; rather, it indicates a strong direction of association that requires confirmation in a larger sample. Although the three-class NPRS solution converged normally, replicated across random starts, and demonstrated strong classification performance (entropy = 0.88; APPs = 94.2%–97.1%), the two- and four-class solutions yielded inadmissible parameter estimates. The three-class solution therefore provided the most interpretable representation of the NPRS data in the present sample. However, this should not be interpreted as evidence that exactly three pain-recovery trajectories exist in the broader population of injured workers. The failure of the alternative solutions may reflect limitations related to model specification, sample size, or their interaction, and replication in larger independent samples is needed to evaluate the stability and prevalence of these trajectory patterns. The EQ-5D-5L trajectory solution also warrants cautious interpretation because model stability required residual variances to be constrained to be equal across time within each class. Although this specification produced a stable and clinically interpretable two-class solution, it assumes comparable unexplained within-class variability at baseline, 4 weeks, and 8 weeks. This assumption may have oversimplified the underlying trajectory structure, particularly during the initial phase of rehabilitation when participants may have shown greater individual heterogeneity in HRQoL that regressed towards the mean nearer the end of the PoC. Consequently, the two identified trajectories should be viewed as a parsimonious representation of the observed data rather than evidence that only two distinct HRQoL recovery patterns exist. Replication in larger samples with sufficient information to support less constrained models is needed to determine whether additional or more heterogeneous recovery trajectories can be identified.

Despite some need for cautious interpretation, a story has emerged across the three companion studies that appear to hold potential value for clinical decision-making, though any such value has yet to be proven. From the first study [22] either the TIDS or the EQ-5D-5L could serve as initial screening tools to identify acutely injured workers that can either be expected to recover well with minimal intervention or those who would benefit from additional workup. Following that, by administering the seven-axis framework questionnaires and using the algorithm derived from the second study in this series [cite when available], estimations of recovery likelihood can be strengthened, and initial treatment directions can start to form. Those who fall into the lowest risk categories on both, which on cross-tabulation includes about 10% of the injured workers in our study, might be expected to respond well to hands-off conservative intervention, including advice, education, and watchful waiting while avoiding over-treatment. The others would benefit from further workup, where the class structure offers initial guidance: for example, more Mood-Dominant phenotypes may benefit from approaches targeting maladaptive cognitions or emotional wellbeing (e.g.[38,39],) while those with a more neurosensory-dominant presentation may be more suited to sensorimotor retraining (e.g., [40]) or pharmaceutical intervention for neuropathic pain. Whether these factors lead to improved decisions on intervention and better clinical efficiency are questions for future research.

Some of the limitations to interpretation have already been addressed. Others include the entirely self-reported nature of the data for this study. This is both a strength and a weakness in that it is, by definition, patient-centred (injured workers provided all data themselves save for the administrative outcome) but increases the potential for misinterpreting some questions or lack of attention when completing. The attrition rate falls just below the typical standard of 80% retention for good prognostic research (e.g.[41],), and it is possible that those who were lost were in some way systematically different from completers in ways that were not captured. The 8-week follow-up also coincided with completion of the initial MSK PoC and therefore captures early rehabilitation response rather than sustained recovery. Recovery status may continue to change after program completion, particularly for workers with incomplete recovery at 8 weeks, and the stability of both trajectory membership and phenotype–outcome associations beyond this period is unknown. Longer-term follow-up at 6 and 12 months will therefore be important to determine whether baseline phenotyping predicts persistent symptoms, sustained return to work, and subsequent healthcare needs. Finally, we did not attempt to control or capture visit-to-visit specifics of interventions provided by the physiotherapists, though the guidelines of the MSK PoC meant the treatment was moderately standardized. The PoC sets expectations around the number of visits and the types of treatment provided, while allowing for some flexibility based on clinician reasoning. All participants in this study received physiotherapy care, typically on a one- to two-visit-per-week schedule and received some combination of supervised exercise, hands-on manual therapy, pain management modalities such as heat, ice, or transcutaneous electrical stimulation (TENS), and advice and education related to home management. The PoC provides few provisions for treatment outside of these guidelines, unless approved for an alternative level of care, which would have then excluded them from our study.

## Conclusion

Among workers with recent work-related musculoskeletal injuries receiving the Ontario WSIB Musculoskeletal Program of Care, baseline multidimensional self-report phenotypes were associated with distinct short-term pain, health-related quality of life, return to work, and administrative outcomes, but not with self-rated global recovery. The Localized/Resilient phenotype generally showed the most favorable outcomes, whereas the Complex/Central phenotype showed the highest odds of poor outcomes, although estimates for this small subgroup were imprecise. These findings support prospective validation of multidimensional phenotyping as a risk-stratification approach, including evaluation of its longer-term prognostic value and clinical utility beyond the 8-week rehabilitation period.

## Supporting information

S1 Table Results of Latent Growth Curve Analyses (LGCA) of 1–4 class solutions using EuroQoL-5D (EQ-5D-5L) scores at three time points: intake, 1- and 2-month follow-up. AIC = Akaike Information Criterion; adj.BIC = sample size-adjusted Bayesian Information Criterion; APP = Average Posterior Probabilities; LMR-adj.LRT = Lo-Mendell-Rubin adjusted Likelihood Ratio Test.
(DOCX)

## References

[1] Global, regional, and national burden of other musculoskeletal disorders, 1990-2020, and projections to 2050: a systematic analysis of the Global Burden of Disease Study 2021. Lancet Rheumatol. 2023;5(11):e670–82. 10.1016/S2665-9913(23)00232-1 PMC10620749 37927903

[2] Thanh NX, Tanguay RL, Manhas KJP, Kania-Richmond A, Kashuba S, Geyer T, et al. Economic burden of chronic pain in Alberta, Canada. PLoS One. 2022;17(8):e0272638. doi: 10.1371/journal.pone.0272638 35960750 PMC9374207

[3] Santos W, Rojas C, Isidoro R, Lorente A, Dias A, Mariscal G, et al. Return to Work After Work-Related Injuries: A Systematic Review and Meta-Analysis of Incidence and Determinants. J Clin Med. 2025;14(12):4343. doi: 10.3390/jcm14124343 40566086 PMC12194668

[4] Walton DM, Macdermid JC, Taylor T. What does “recovery” mean to people with neck pain? Results of a descriptive thematic analysis. Open Orthop J. 2013;7:420–7. doi: 10.2174/1874325001307010420 24115969 PMC3793580

[5] Cullen KL, Irvin E, Collie A, Clay F, Gensby U, Jennings PA, et al. Effectiveness of Workplace Interventions in Return-to-Work for Musculoskeletal, Pain-Related and Mental Health Conditions: An Update of the Evidence and Messages for Practitioners. J Occup Rehabil. 2018;28(1):1–15. doi: 10.1007/s10926-016-9690-x 28224415 PMC5820404

[6] Workplace Safety and Insurance Board. Musculoskeletal (MSK) program of care training slides. Toronto, Ontario: Workplace Safety and Insurance Board. 2023. https://www.wsib.ca/en/document/musculoskeletal-msk-program-care-training-slides

[7] Lee JY, Walton DM, Tremblay P, May C, Millard W, Elliott JM, et al. Defining pain and interference recovery trajectories after acute non-catastrophic musculoskeletal trauma through growth mixture modeling. BMC Musculoskelet Disord. 2020;21(1):615. doi: 10.1186/s12891-020-03621-7 32943021 PMC7495896

[8] Ogollah RO, Konstantinou K, Stynes S, Dunn KM. Determining One-Year Trajectories of Low-Back-Related Leg Pain in Primary Care Patients: Growth Mixture Modeling of a Prospective Cohort Study. Arthritis Care Res (Hoboken). 2018;70(12):1840–8. doi: 10.1002/acr.23556 29575673

[9] Walton DM, Bobos P, MacDermid JC. Latent Profile Analysis of Canadian Military Veterans With Chronic Pain Identifies 5 Meaningful Classes Through Self-Report Measures. J Pain. 2024;25(8):104517. doi: 10.1016/j.jpain.2024.03.013 38609027

[10] Lee JY, Walton DM. Latent profile analysis of blood marker phenotypes and their relationships with clinical pain and interference reports in people with acute musculoskeletal trauma. Can J Pain. 2021;5(1):30–42. doi: 10.1080/24740527.2020.1870102 33987522 PMC7951147

[11] Kongsted A, Kent P, Axen I, Downie AS, Dunn KM. What have we learned from ten years of trajectory research in low back pain?. BMC Musculoskeletal Disorders. 2016;17(1):220. doi: 10.1186/s12891-016-1160-5 PMC4875630 27209166

[12] Downie AS, Hancock MJ, Rzewuska M, Williams CM, Lin C-WC, Maher CG. Trajectories of acute low back pain: a latent class growth analysis. Pain. 2016;157(1):225–34. doi: 10.1097/j.pain.0000000000000351 26397929

[13] Carroll LJ, Lis A, Weiser S, Torti J. How Well Do You Expect to Recover, and What Does Recovery Mean, Anyway? Qualitative Study of Expectations After a Musculoskeletal Injury. Phys Ther. 2016;96(6):797–807. doi: 10.2522/ptj.20150229 26586855

[14] Middlebrook N, Heneghan NR, Moffatt M, Silvester L, Falla D, Rushton AB, et al. Experiences, views and perceptions of recovery following musculoskeletal trauma of patients and physiotherapists: a qualitative study. PLoS One. 2025;20(5):e0323575. doi: 10.1371/journal.pone.0323575 40435443 PMC12119110

[15] Middlebrook N, Heneghan NR, Moffatt M, Silvester L, Falla D, Rushton AB, et al. Lived experiences of recovery following musculoskeletal trauma 6 months following injury in the UK: a qualitative study. BMJ Open. 2025;15(11):e108425. doi: 10.1136/bmjopen-2025-108425 41263933 PMC12636951

[16] Carrière JS, Donayre Pimentel S, Bou Saba S, Boehme B, Berbiche D, Coutu M-F, et al. Recovery expectations can be assessed with single-item measures: findings of a systematic review and meta-analysis on the role of recovery expectations on return-to-work outcomes after musculoskeletal pain conditions. Pain. 2023;164(4):e190–206. doi: 10.1097/j.pain.0000000000002789 36155605 PMC10026834

[17] Cancelliere C, Donovan J, Stochkendahl MJ, Biscardi M, Ammendolia C, Myburgh C, et al. Factors affecting return to work after injury or illness: best evidence synthesis of systematic reviews. Chiropr Man Therap. 2016;24(1):32. doi: 10.1186/s12998-016-0113-z 27610218 PMC5015229

[18] Walton DM, Carroll LJ, Kasch H, Sterling M, Verhagen AP, Macdermid JC, et al. An overview of systematic reviews on prognostic factors in neck pain: results from the International Collaboration on Neck Pain (ICON) Project. Open Orthop J. 2013;7:494–505. doi: 10.2174/1874325001307010494 24115971 PMC3793581

[19] Walton DM, Macdermid JC, Giorgianni AA, Mascarenhas JC, West SC, Zammit CA. Risk factors for persistent problems following acute whiplash injury: update of a systematic review and meta-analysis. J Orthop Sports Phys Ther. 2013;43(2):31–43. doi: 10.2519/jospt.2013.4507 23322093

[20] Meisingset I, Vasseljen O, Vøllestad NK, Robinson HS, Woodhouse A, Engebretsen KB, et al. Novel approach towards musculoskeletal phenotypes. Eur J Pain. 2020;24(5):921–32. doi: 10.1002/ejp.1541 32040225

[21] Aasdahl L, Granviken F, Meisingset I, Woodhouse A, Evensen KAI, Vasseljen O. Recovery trajectories in common musculoskeletal complaints by diagnosis contra prognostic phenotypes. BMC Musculoskelet Disord. 2021;22(1):455. doi: 10.1186/s12891-021-04332-3 34011349 PMC8132354

[22] Bayattork M, Abadi SS, Carter T, Cooper L, Walton DM. Social determinants of pain, distress, and quality of life in injured workers: A cross-sectional and longitudinal analysis of patient-reported outcomes. PLoS One. 2026;21(4):e0346697. doi: 10.1371/journal.pone.0346697 42030263 PMC13108777

[23] Cuschieri S. The STROBE guidelines. Saudi J Anaesth. 2019;13(Suppl 1):S31–4. doi: 10.4103/sja.SJA_543_18 30930717 PMC6398292

[24] Bennett MI, Smith BH, Torrance N, Potter J. The S-LANSS score for identifying pain of predominantly neuropathic origin: validation for use in clinical and postal research. J Pain. 2005;6(3):149–58. doi: 10.1016/j.jpain.2004.11.007 15772908

[25] Walton DM, Marsh J. The Multidimensional Symptom Index: A new patient-reported outcome for pain phenotyping, prognosis and treatment decisions. Eur J Pain. 2018;22(7):1351–61. doi: 10.1002/ejp.1224 29635812

[26] McWilliams LA, Kowal J, Wilson KG. Development and evaluation of short forms of the Pain Catastrophizing Scale and the Pain Self-efficacy Questionnaire. Eur J Pain. 2015;19(9):1342–9. doi: 10.1002/ejp.665 25766681

[27] Löwe B, Wahl I, Rose M, Spitzer C, Glaesmer H, Wingenfeld K, et al. A 4-item measure of depression and anxiety: validation and standardization of the Patient Health Questionnaire-4 (PHQ-4) in the general population. J Affect Disord. 2010;122(1–2):86–95. doi: 10.1016/j.jad.2009.06.019 19616305

[28] Walton DM, Nazari G, Bobos P, MacDermid JC. Exploratory and confirmatory factor analysis of the new region-generic version of Fremantle Body Awareness-General Questionnaire. PLoS One. 2023;18(3):e0282957. doi: 10.1371/journal.pone.0282957 36947566 PMC10032497

[29] Scheim AI, Bauer GR. The intersectional discrimination index: development and validation of measures of self-reported enacted and anticipated discrimination for intercategorical analysis. Soc Sci Med. 2019;226:225–35. doi: 10.1016/j.socscimed.2018.12.016 30674436

[30] Herdman M, Gudex C, Lloyd A, Janssen M, Kind P, Parkin D, et al. Development and preliminary testing of the new five-level version of EQ-5D (EQ-5D-5L). Qual Life Res. 2011;20(10):1727–36. doi: 10.1007/s11136-011-9903-x 21479777 PMC3220807

[31] Linton SJ, Nicholas M, MacDonald S. Development of a short form of the Örebro Musculoskeletal Pain Screening Questionnaire. Spine (Phila Pa 1976). 2011;36(22):1891–5. doi: 10.1097/BRS.0b013e3181f8f775 21192286

[32] Traeger A, McAuley JH. STarT Back Screening Tool. J Physiother. 2013;59(2):131. doi: 10.1016/S1836-9553(13)70170-X 23663803

[33] Vigdal ØN, Flugstad S, Storheim K, Killingmo RM, Grotle M. Predictive validity of the STarT Back screening tool among older adults with back pain. Eur J Pain. 2024;28(9):1559–70. doi: 10.1002/ejp.2281 38752601

[34] Forsbrand MH, Grahn B, Hill JC, Petersson IF, Post Sennehed C, Stigmar K. Can the STarT Back Tool predict health-related quality of life and work ability after an acute/subacute episode with back or neck pain? A psychometric validation study in primary care. BMJ Open. 2018;8(12):e021748. doi: 10.1136/bmjopen-2018-021748 30580256 PMC6318523

[35] Heikkala E, Oura P, Ruokolainen O, Ala-Mursula L, Linton SJ, Karppinen J. The Örebro Musculoskeletal Pain Screening Questionnaire-Short Form and 2-year follow-up of registered work disability. Eur J Public Health. 2023;33(3):442–7. doi: 10.1093/eurpub/ckad079 37192056 PMC10234666

[36] Kwon J-Y, Sawatzky R, Baumbusch J, Lauck S, Ratner PA. Growth mixture models: a case example of the longitudinal analysis of patient-reported outcomes data captured by a clinical registry. BMC Med Res Methodol. 2021;21(1):79. doi: 10.1186/s12874-021-01276-z 33882863 PMC8058975

[37] Yan J, Xie S, Johnson JA, Pullenayegum E, Ohinmaa A, Bryan S, et al. Canada population norms for the EQ-5D-5L. Eur J Health Econ. 2024;25(1):147–55. doi: 10.1007/s10198-023-01570-1 36828968

[38] Aabedi A, Wang V, Fraix MP, Agrawal DK. Psychological Treatment in the Management of Pain following Musculoskeletal Injury. J Orthop Sports Med. 2025;7(1):162–8. doi: 10.26502/josm.511500191 40297561 PMC12037177

[39] Rijsdijk M, Smits HM, Azizoglu HR, Brugman S, van de Burgt Y, van Charldorp TC, et al. Identifying patient subgroups in the heterogeneous chronic pain population using cluster analysis. J Pain. 2025;28:104792. doi: 10.1016/j.jpain.2025.104792 39855407

[40] Bagg MK, Wand BM, Cashin AG, Lee H, Hübscher M, Stanton TR, et al. Effect of Graded Sensorimotor Retraining on Pain Intensity in Patients With Chronic Low Back Pain: A Randomized Clinical Trial. JAMA. 2022;328(5):430–9. doi: 10.1001/jama.2022.9930 35916848 PMC9346551

[41] Heikkala E, Oura P, Ruokolainen O, Ala-Mursula L, Linton SJ, Karppinen J. The Örebro Musculoskeletal Pain Screening Questionnaire-Short Form and 2-year follow-up of registered work disability. Eur J Public Health. 2023;33(3):442–7. doi: 10.1093/eurpub/ckad079 37192056 PMC10234666

