# Supplementary material for: Defining and predicting outcomes of a work-injuries rehabilitation program through multivariate baseline phenotypes and longitudinal modeling
Source: PLoS One. 2026 Nov 4;21(11):e0362083. doi: 10.1371/journal.pone.0362083 (PMC13734445; doi:10.1371/journal.pone.0362083)
Supplement: Supplementary file 1 [file pone.0362083.s001.docx]

|  | **AIC/adj. BIC** | **Entropy** | **APP** | **Proportions** | **LMR-adj.LRT (p)** |
| --- | --- | --- | --- | --- | --- |
| 1 class | -399.0/-400.9 | 1.00 | 100% | 100% | N/A |
| 2 class | -446.7/-449.3 | 0.91 | 90.1% to 98.8% | 1: 15.1%  2: 81.9% | 50.36 (0.46) |
| 2 class, time-based residuals fixed | -434.9/-437.0 | 0.93 | 96.5% to 98.3% | 1: 86.2%  2: 13.8% | 49.87 (0.01) |
| 3 class | -462.5/-465.8 | 0.93 | 94.9% to 97.4% | 1: 6.5%  2: 14.5%  3: 79.0% | 20.41 (<0.01) |
| 3 class, slope variance constrained to zero | -464.5/-467.4 | 0.89 | 88.1% to 96.6% | 1: 8.0%  2: 75.4%  3: 16.7% | 31.76 (0.21) |
| 3 class, time-based residuals fixed | -449.1/-452.0 | 0.86 | 83.8% to 96.8% | 1: 69.6%  2: 13.0%  3: 17.4% | 18.96 (0.28) |
| 4 class | -472.8/-476.9 | 0.90 | 88.4% to 95.8% | 1: 72.5%  2: 6.5%  3: 6.5%  4: 14.5% | 15.29 (0.34) |
| 4 class, time-based residuals fixed | No convergence | NA | NA | NA | NA |

TABLE S1. Results of Latent Growth Curve Analyses (LGCA) of 1-4 class solutions using EuroQoL-5D (EQ-5D-5L) scores at three time points: intake, 1- and 2-month follow-up. AIC = Akaike Information Criterion; adj.BIC = sample size-adjusted Bayesian Information Criterion; APP = Average Posterior Probabilities; LMR-adj.LRT = Lo-Mendell-Rubin adjusted Likelihood Ratio Test.
