# Supplementary material for: Defining and predicting outcomes of a work-injuries rehabilitation program through multivariate baseline phenotypes and longitudinal modeling
Source: PLoS One. 2026 Nov 4;21(11):e0362083. doi: 10.1371/journal.pone.0362083 (PMC13734445; doi:10.1371/journal.pone.0362083)
Supplement: Supplementary file 2 [file pone.0362083.s002.docx]

**General Comments**

The authors address a relevant and generally well-justified topic. The manuscript evaluates a considerable number of outcomes, which provides a comprehensive perspective on the research question. However, the large amount of information also increases the complexity and length of the manuscript, making some methodological aspects and results difficult to follow at times. Nevertheless, I believe the manuscript has good potential. The comments below are intended to help improve the clarity, organization, and interpretation of the information presented.

**Introduction**

1. The authors provide a clear description of the problem addressed in the Introduction, particularly regarding the variability in individual responses during the rehabilitation process. However, limited information is provided regarding the rehabilitation programs currently recommended or commonly implemented in this context. Although I understand that this is not the primary focus of the manuscript, a brief description of the rehabilitation approaches associated with these heterogeneous responses could provide useful context for readers and strengthen the rationale for the study.

**Methods**

1. The manuscript would benefit from a more detailed description of the rehabilitation program implemented in the study. Although the authors mention the main practices included in the intervention, additional information regarding its structure and implementation would improve reproducibility. I recognize that the primary focus of the study is to understand the variability in responses; nevertheless, readers should have sufficient information about the rehabilitation program from which these responses were derived. I therefore encourage the authors to provide additional details that would allow the intervention to be adequately understood and potentially replicated.
2. Some methodological aspects are described only briefly, with the authors referring readers to a previous study that has not yet been published for additional details. This may limit the reader's ability to fully evaluate the methodology of the present study, as the referenced information is currently unavailable. I encourage the authors to consider providing the essential methodological information directly in the present manuscript. Alternatively, references to the unpublished manuscript could be reconsidered when they serve as the primary source for methodological details necessary to understand or reproduce the study.
3. The manuscript could benefit from the inclusion of a figure illustrating the study design and assessment timeline. In particular, the measures could be presented in a schematic figure indicating the time points at which the “trajectory measures” and “point outcomes” were obtained. Such a figure could facilitate understanding of the study procedures and provide readers with a clearer overview of when each assessment was performed.
4. Regarding sample size, I suggest that the authors explicitly describe the sampling strategy used to recruit participants (e.g., non-probability or convenience sampling). Although it appears clear that no formal sample size calculation was performed, the sampling approach itself is not sufficiently described. Providing this information would improve methodological transparency.

**Results**

1. A similar consideration applies to the initial part of the Results section, particularly the subsection entitled “Fidelity and Data Cleaning.” The inclusion of a participant flowchart could provide a clearer and more intuitive representation of participant progression throughout the study, including losses to follow-up and the corresponding reasons. I recognize that journal restrictions regarding the number of figures and tables may influence how this information can be presented. Therefore, the authors may consider this suggestion according to the journal's formatting requirements and available space.
2. I suggest reconsidering the placement of the information regarding “Point Outcomes,” which is currently presented in the Statistical Analysis section. This information appears to describe how the outcomes were defined or assessed and, therefore, may be more appropriately included in the Measures section. This change could improve the organization and logical flow of the Methods section.
3. Figures 1 and 2 would benefit from more detailed legends to facilitate interpretation without requiring readers to repeatedly consult the main text. In addition, I encourage the authors to reconsider whether presenting the same findings in both figures (Figures 1 and 2) and a table (Table 2) is necessary. If these elements present essentially the same results, selecting the format that communicates the findings most effectively could reduce redundancy and improve the overall conciseness of the manuscript.
4. The classifications “Rapid Recovery,” “Incomplete Recovery,” and “No Recovery” appear to be introduced primarily in the Results section. For methodological clarity, I suggest describing the classification approach and the criteria used to define these categories in the Methods section before presenting the corresponding results. This would allow readers to understand how participants or outcomes were classified before interpreting the findings.
5. Several abbreviations are used throughout the tables. I recommend defining all abbreviations in the corresponding table legends or footnotes so that each table can be interpreted independently from the main text.

**Discussion**

1. At the beginning of the Discussion, findings from previous studies (e.g., “Study One”) are presented in close proximity to the findings of the current study. In some instances, this may make it difficult for readers to distinguish between results derived from the present analysis and those reported previously. I suggest emphasizing the main findings of the current study at the beginning of the Discussion and subsequently using findings from previous studies to contextualize, compare, and interpret the present results. This consideration may also be relevant to other parts of the Discussion. Maintaining the findings of the current manuscript as the central focus throughout the section could improve clarity and strengthen the interpretation of the study.

**Conclusion**

1. The Conclusion could be more concise and focused, as some statements appear to reiterate results already described in previous sections of the manuscript. I encourage the authors to provide a broader synthesis of the main findings rather than restating individual results. The Conclusion could emphasize the most relevant overall findings and their implications. In addition, I suggest framing the conclusions more explicitly in relation to the specific rehabilitation program evaluated in this study, as the observed findings and response patterns should be interpreted within the context of this particular rehabilitation model.
