# Supplementary material for: Defining and predicting outcomes of a work-injuries rehabilitation program through multivariate baseline phenotypes and longitudinal modeling
Source: PLoS One. 2026 Nov 4;21(11):e0362083. doi: 10.1371/journal.pone.0362083 (PMC13734445; doi:10.1371/journal.pone.0362083)
Supplement: Supplementary file 3 [file pone.0362083.s004.docx]

Response to the Academic Editor and Reviewers

Dear Editor,

We sincerely thank the Academic Editor and the three reviewers for their careful evaluation of our manuscript and for the constructive recommendations provided. We have carefully considered every comment and have substantially revised the manuscript to improve methodological transparency, statistical interpretation, organization, reproducibility, and clarity. All changes will be identified by page and line number in the final revised manuscript.

Response to Journal Requirements:

When submitting your revision, we need you to address these additional requirements.

1.Please ensure that your manuscript meets PLOS ONE's style requirements, including those for file naming.

**Response: We have reviewed the manuscript against the current PLOS ONE formatting and file-naming requirements and have revised the manuscript and accompanying files accordingly. We have also undertaken a complete formatting review to ensure consistent headings, tables, abbreviations, figures, references, and supporting information captions.**

2. Please note that PLOS One has specific guidelines on code sharing for submissions in which author-generated code underpins the findings in the manuscript. In these cases, all author-generated code must be made available without restrictions upon publication of the work. Please review our guidelines at <https://journals.plos.org/plosone/s/materials-and-software-sharing#loc-sharing-code> and ensure that your code is shared in a way that follows best practice and facilitates reproducibility and reuse.

**RESPONSE: There may be a subtle nuance to this response, because we wouldn’t consider the code ‘author-generated’ in that MPlus user manuals provide the code-line prompts for the types of analyses here. In our case the GMM technique is called with**

**ANALYSIS: Type = Mixture**

**which is the baked-in software command. The only real author-generated specifications here are number of random starts (1000 250 in our case) and STITERATIONS (20) which is more of a trial-and-error thing to ensure model convergence. For constraining the variance across time, again this is MPlus code not what I would want to claim as our own code, e.g,:**

**i s | T0@0 T1@1 T2@2**

**indicating we want to constrain time-dependent variance in intercept and slope independently for each of times 0, 1, and 2. This was only required for the EQ5D-5L analysis but not the NPRS analysis which converged on 3 latent trajectories without requiring any additional constraints, which we’ve made clear in the manuscript. So I wouldn’t want to claim any of this as ‘our code’, rather it’s our application of MPlus code with any constraints informed by the nature of the data. In this way it would be conceptually similar to someone copy/pasting the Syntax from SPSS based on the buttons they pressed in that software, the biggest difference being that SPSS uses a graphic interface while MPlus uses more of a command line interface. Posting this as author-generated code feels disingenuous though it certainly would not be hard (a few lines) if that is required, as long as we can include appropriate caveats that we are not claiming ownership of MPlus language.**

3. We note that the grant information you provided in the ‘Funding Information’ and ‘Financial Disclosure’ sections do not match.

When you resubmit, please ensure that you provide the correct grant numbers for the awards you received for your study in the ‘Funding Information’ section.

**WSIB does not use a typical structure for grant numbers, rather they use an internal value of the first 4 letters of the PIs surname plus the year of the award, so in this case that was WALT2022. These are also not posted publicly, so I typically indicate ‘N/A’ in the grant number section for this work. Nonetheless, at this point I don’t think there’s any longer a compelling reason to keep that private so the number has been added to the Funding Information and Financial Disclosures section.**

4. Thank you for stating the following financial disclosure:

“Ontario Workplace Safety and Insurance Board Research Grant awarded to D. Walton, held and managed by the Western University Research Finance Office.”

Please state what role the funders took in the study.  If the funders had no role, please state: "The funders had no role in study design, data collection and analysis, decision to publish, or preparation of the manuscript."

If this statement is not correct you must amend it as needed.

Please include this amended Role of Funder statement in your cover letter; we will change the online submission form on your behalf.fo

**RESPONSE: We can confirm that the statement as provided here is accurate. This has been addressed in the cover letter and can be updated in the online form.**

5. Thank you for stating the following in the Competing Interests section:

“I have read the journal's policy and the authors of this manuscript have the following competing interests: D. Walton is co-author of a text book published in 2020 (pub: Singing Dragon Press) in which the seven-axis framework from which the baseline variables used in this study was derived. The results of this study have no influence on the contents of that book.”

We note that one or more of the authors are employed by a commercial company: “CBI Health Group Inc., Ontario, Canada and Singing Dragon Press”

1. Please provide an amended Funding Statement declaring this commercial affiliation, as well as a statement regarding the Role of Funders in your study. If the funding organization did not play a role in the study design, data collection and analysis, decision to publish, or preparation of the manuscript and only provided financial support in the form of authors' salaries and/or research materials, please review your statements relating to the author contributions, and ensure you have specifically and accurately indicated the role(s) that these authors had in your study. You can update author roles in the Author Contributions section of the online submission form.

Please also include the following statement within your amended Funding Statement.

“The funder provided support in the form of salaries for authors “DW and TC”, but did not have any additional role in the study design, data collection and analysis, decision to publish, or preparation of the manuscript. The specific roles of these authors are articulated in the ‘author contributions’ section.”

If your commercial affiliation did play a role in your study, please state and explain this role within your updated Funding Statement.

2. Please also provide an updated Competing Interests Statement declaring this commercial affiliation along with any other relevant declarations relating to employment, consultancy, patents, products in development, or marketed products, etc.

Within your Competing Interests Statement, please confirm that this commercial affiliation does not alter your adherence to all PLOS ONE policies on sharing data and materials by including the following statement: "This does not alter our adherence to  PLOS ONE policies on sharing data and materials.” (as detailed online in our guide for authors <http://journals.plos.org/plosone/s/competing-interests>) . If this adherence statement is not accurate and  there are restrictions on sharing of data and/or materials, please state these. Please note that we cannot proceed with consideration of your article until this information has been declared.

Please include both an updated Funding Statement and Competing Interests Statement in your cover letter. We will change the online submission form on your behalf.

6. Thank you for stating the following in the Acknowledgments Section of your manuscript:

“We thank the staff and clients at CBI Health Inc. in Ontario for their contributions to the 522 success of this study. We also thank Lynn Cooper from the Canadian Injured Workers 523 Alliance for her early input in shaping the study concept and helping ensure its 524 relevance to the lived experiences and rehabilitation priorities of injured workers.”

We note that you have provided funding information that is not currently declared in your Funding Statement. However, funding information should not appear in the Acknowledgments section or other areas of your manuscript. We will only publish funding information present in the Funding Statement section of the online submission form.

Please remove any funding-related text from the manuscript and let us know how you would like to update your Funding Statement. Currently, your Funding Statement reads as follows:

“Ontario Workplace Safety and Insurance Board Research Grant awarded to D. Walton, held and managed by the Western University Research Finance Office.”

Please include your amended statements within your cover letter; we will change the online submission form on your behalf.

**RESPONSE: We have addressed this item at length in the Cover Letter so will not repeat that here. It seems as though the Editor/reviewer may be seeing something in this Acknowledgement that is unintended. No funding, whether direct or in-kind, is implicated by this statement, which we are using largely as a public thank you to those who helped make the study a success.**

7. Thank you for uploading your study's underlying data set. Unfortunately, the repository you have noted in your Data Availability statement does not qualify as an acceptable data repository according to PLOS's standards.

At this time, please upload the minimal data set necessary to replicate your study's findings to a stable, public repository (such as figshare or Dryad) and provide us with the relevant URLs, DOIs, or accession numbers that may be used to access these data. For a list of recommended repositories and additional information on PLOS standards for data deposition, please see [https://journals.plos.org/plosone/s/recommended-repositories](https://track.editorialmanager.com/CL0/https:%2F%2Fjournals.plos.org%2Fplosone%2Fs%2Frecommended-repositories/1/010f019fcaed4680-434bc580-baf0-4638-98ff-ca7ba91165f5-000000/7zsR_V7nYp3l8V7mUDyZ1Xjt9dyFLV4O9bwsDlibAic=258).

**RESPONSE: Curiously the Borealis repository has been accepted on prior manuscripts submitted to PLoS One, but nevertheless we have now also linked to those through the project’s OSF page at doi** [**10.17605/OSF.IO/XVUQ5**](https://doi.org/10.17605/OSF.IO/XVUQ5)**. OSF appears to be listed on the journal’s list of approved data repositories.**

8. Please include your full ethics statement in the ‘Methods’ section of your manuscript file. In your statement, please include the full name of the IRB or ethics committee who approved or waived your study, as well as whether or not you obtained informed written or verbal consent. If consent was waived for your study, please include this information in your statement as well.

**Response: We have expanded the ethics statement in the Methods section to provide the full name of the approving ethics board, the protocol number, and the consent procedure.**

9. We notice that your supplementary figures are uploaded with the file type 'Figure'. Please amend the file type to 'Supporting Information'. Please ensure that each Supporting Information file has a legend listed in the manuscript after the references list.

**RESPONSE: The Supplemental Table (S1) has now been uploaded with the File Type of supporting information.**

10. If the reviewer comments include a recommendation to cite specific previously published works, please review and evaluate these publications to determine whether they are relevant and should be cited. There is no requirement to cite these works unless the editor has indicated otherwise.

**Response: We evaluated all references relevant to the reviewers’ comments for their scientific relevance and have incorporated appropriate references where they materially support the statements made in the manuscript.**

Reviewer's Responses to Questions

Comments to the Author

1. Is the manuscript technically sound, and do the data support the conclusions?
The manuscript must describe a technically sound piece of scientific research with data that supports the conclusions. Experiments must have been conducted rigorously, with appropriate controls, replication, and sample sizes. The conclusions must be drawn appropriately based on the data presented.

Reviewer #1: Yes

Reviewer #2: Yes

Reviewer #3: Partly

2. Has the statistical analysis been performed appropriately and rigorously?

Reviewer #1: Yes

Reviewer #2: Yes

Reviewer #3: Yes

3. Have the authors made all data underlying the findings in their manuscript fully available?

The [PLOS Data policy](http://track.editorialmanager.com/CL0/http:%2F%2Fwww.plosone.org%2Fstatic%2Fpolicies.action%23sharing/1/010f019fcaed4680-434bc580-baf0-4638-98ff-ca7ba91165f5-000000/xx2N_9XBoXnTcmacQ17QnDvjoPveHQZEi5amy9t0HWk=258) requires authors to make all data underlying the findings described in their manuscript fully available without restriction, with rare exception (please refer to the Data Availability Statement in the manuscript PDF file). The data should be provided as part of the manuscript or its supporting information, or deposited to a public repository. For example, in addition to summary statistics, the data points behind means, medians and variance measures should be available. If there are restrictions on publicly sharing data—e.g. participant privacy or use of data from a third party—those must be specified.

Reviewer #1: Yes

Reviewer #2: No

Reviewer #3: Yes

4. Is the manuscript presented in an intelligible fashion and written in standard English?

PLOS ONE does not copyedit accepted manuscripts, so the language in submitted articles must be clear, correct, and unambiguous. Any typographical or grammatical errors should be corrected at revision, so please note any specific errors here.

Reviewer #1: Yes

Reviewer #2: Yes

Reviewer #3: Yes

5. Review Comments to the Author

Please use the space provided to explain your answers to the questions above. You may also include additional comments for the author, including concerns about dual publication, research ethics, or publication ethics. (Please upload your review as an attachment if it exceeds 20,000 characters)

Reviewer #1: Dear Authors,
As a reviewer for this manuscript, I provide below my considerations and suggestions for improvement to strengthen the article "Defining and predicting outcomes of a work-injuries rehabilitation program through multivariate baseline phenotypes and longitudinal modeling" for potential publication in PLOS One. The study addresses a highly relevant clinical topic—the phenotyping of patients to predict recovery trajectories in injured workers—and employs robust, advanced statistical techniques.

The following points warrant attention:
1. Statistical Robustness and Sample Size
While the use of Growth Mixture Modeling (GMM) and Latent Profile Analysis (LPA) is a strength of this work, the sample size (N = 145) is on the lower end of the recommended range for these techniques, which typically require between 100 to 300 participants to ensure class stability.
• Suggestion: I recommend expanding the discussion on how the sample size may have influenced model convergence, particularly for pain (NPRS), where 2- and 4-class models were found to be inadmissible.

**Response: We agree that, although our sample size is within the range reported in previous GMM studies, it is relatively modest for mixture modeling and may have affected the stability of alternative class solutions, particularly when estimating smaller latent classes.**

**We have therefore revised the manuscript to more explicitly acknowledge the sample size dependence of the NPRS trajectory solution. In the present analysis, the two- and four-class NPRS models produced inadmissible parameter estimates, whereas the three-class model converged normally, replicated across random starts, and demonstrated good classification indices (entropy = 0.88; average posterior probabilities = 94.2%–97.1%). We have clarified that the successful convergence and good fit of the three-class model support its interpretation within the present sample but do not establish that three trajectories represent the definitive underlying population structure. The failure of the alternative models may reflect limitations in model specification, available sample information, or both.**

**We have revised the Sample Size subsection of the Methods (lines 260 to 267) and the Discussion (lines 484 to 492) accordingly and now emphasize the need for replication in larger independent samples.**

2. Confidence Intervals and Stability of the "Complex/Central" Group
The results indicate an extremely high Odds Ratio (OR) of 150.00 for the "Complex/Central" group in predicting "No Improvement" trajectories in quality of life, but with a very wide 95% Confidence Interval (18.53–1214.16).
• Suggestion: It is imperative to emphasize in the discussion that these values are effect estimates and may be unstable due to the small number of participants in this specific phenotype (representing only 8.5% of the sample). Please avoid definitive conclusions based on such high point estimates without clearly noting the statistical imprecision.

**Response: We agree that the very large odds ratio observed for the Complex/Central phenotype should not be interpreted as a precise estimate of effect magnitude. This phenotype represented the smallest baseline subgroup, resulting in sparse comparisons and correspondingly wide confidence intervals. In particular, although the association between the Complex/Central phenotype and the EQ-5D-5L No Recovery trajectory yielded an OR of 150.00, the 95% confidence interval was very wide (18.53–1214.16), indicating substantial statistical imprecision.**

**We have therefore revised the Results and Discussion to explicitly distinguish the direction and apparent strength of the association from the precision of the estimated effect. The revised manuscript now includes greater emphasis on the wide range of point estimates (Results lines 370 to 373) and the Discussion section includes greater emphasis that these large point estimates should be interpreted cautiously and require confirmation in larger independent samples (lines 476 to 483). We have also added a brief qualification in the Abstract so that the statistical imprecision of estimates involving the small Complex/Central subgroup is clear when the principal findings are first presented.**

3. Constraints in the Quality of Life Model (EQ-5D-5L)
You mentioned that to achieve model convergence for the quality of life trajectories, it was necessary to constrain residual variances to be equal over time within each class.
• Suggestion: Provide a more detailed explanation in the text (beyond the limitations section) regarding how this constraint might have oversimplified the class structure. Specifically, discuss if it potentially masked individual heterogeneity during the initial phase of rehabilitation.

**Response: We have strengthened the Discussion to acknowledge that this assumption may have oversimplified the trajectory structure, particularly during the initial phase of rehabilitation when individual HRQoL responses may have been more heterogeneous while scores for participants may have regressed towards a mean over the course of the 8-week rehabilitation program (this is a hypothetical at this point, we are unaware of prior empirical evidence to suggest variance decreases in this way though it seems worthy of exploration). We therefore now describe the two-class solution as a parsimonious representation of the observed data rather than evidence that only two HRQoL recovery patterns exist in the underlying population and emphasize the need for replication in larger samples capable of supporting less constrained models (lines 542 to 554 and 492 to 504).**

4. Updating Missing Citations
The manuscript contains several "[cite when available]" placeholders in critical sections, such as the description of input variables for the LPA and the grounding of the screening algorithms.
• Suggestion: Before final submission, these placeholders must be replaced with full references (or indicated as "in press" where applicable). Clarity regarding the "seven-axis framework" is essential for the replicability of the study.

**RESPONSE: Through unfortunate timing on the review end, the reviews for this paper (the third in a series of 3) were returned to us before the reviewers for the second in the series, despite having submitted the latter several months prior. Fortunately the reviews on the second paper were very brief so we were able to turn it around quickly and it has been resubmitted for second review though as we write this response, we still do not have a citation for that. We are confident that, given the minimal revisions on the second paper, it will be accepted and given a doi for online publication well before this third paper is through the next round of reviews, so will be able to update that citation at the typesetting stage of this paper.**

5. Attrition Rate
The retention rate was approximately 77%, which is slightly below the commonly accepted 80% standard for high-quality prognostic studies.
• Suggestion: Briefly provide a comparative analysis between participants who completed the study and those lost to follow-up (regarding demographic characteristics and baseline pain) to rule out potential systematic selection bias.

**RESPONSE: We have run the comparison between participants who provided complete data across all time points and those who did not in terms of age, sex, and baseline NPRS scores and found no significant difference. This has been added to the first paragraph of the Results section (lines 276 to 277).**

6. Detailing the Intervention
The article focuses on prediction but describes the intervention somewhat generically as a "standardized program of care".
• Suggestion: While the treatment is guided by WSIB protocols, adding a more detailed description of the average session frequency (e.g., 1–2 times per week) and specific components (exercise, manual therapy, education) would help the reader understand the context of what the "No Recovery" groups experienced.

**Response: We have expanded the methods using the WSIB program specifications applicable during the study period (lines 144 to155).**

7. Follow-up Period
The study evaluates outcomes up to 8 weeks, which is a short-term window in the context of occupational injuries.
• Suggestion: Discuss whether initial phenotyping might also hold value for predicting long-term outcomes (e.g., 6 or 12 months), as tools like STarT Back are often validated over longer timeframes.

**RESPONSE: The 8-week endpoint was selected because it corresponds to completion of the initial WSIB Musculoskeletal Program of Care; accordingly, it is important to note that the present findings should be interpreted as short-term prognostic associations. We have expanded the Discussion to make this distinction explicit and to place our findings in the context of established risk-screening approaches such as STarT Back and the Örebro questionnaire, which have commonly been evaluated over 6–12 months or longer. We now emphasize that the associations observed in the present study provide a rationale, but not evidence, for longer-term prognostic validity.**

**We have also added a specific recommendation for prospective follow-up at 6 and 12 months to determine whether baseline phenotype membership predicts sustained pain and health-related quality-of-life outcomes, durable return to work, and subsequent healthcare needs.**

In conclusion, this study is innovative in demonstrating that phenotypes derived entirely from brief self-reports (taking about 5 minutes) can effectively stratify risk in acutely injured workers. With these adjustments, the work will make a significant impact on the literature regarding risk-stratified musculoskeletal rehabilitation.

Reviewer #2: Overall, this study investigated whether multidimensional baseline phenotypes based on brief self-reported data can predict early pain and HRQoL trajectories and short-term rehabilitation outcomes among injured workers. The results demonstrate that baseline multivariate phenotyping effectively stratifies patients with distinct rehabilitation prognoses. This is a well-structured and interesting manuscript. Several minor and moderate revisions are suggested to improve the presentation, rigor, and readability of this work.

1. The sentence in Lines 25-28 is overly lengthy and difficult to follow. Please rephrase and simplify it for better readability.

**RESPONSE: We have revised it into two shorter sentences that more clearly distinguish the two study aims: “**To characterize short-term pain and health-related quality-of-life (HRQoL) recovery trajectories in injured workers undergoing standardized musculoskeletal rehabilitation. We also examined whether multidimensional baseline phenotypes derived from brief self-report measures predicted these trajectories and subsequent recovery.”

2. The approximate proportion values (≈55%, 20%, 15%, 10%) in Lines 32-33 are inappropriate for formal reporting. Please provide exact percentages with one decimal place to ensure consistent formatting.

**RESPONSE: We have removed the approximate values and now report the distribution of baseline phenotype membership in the present analytic sample to one decimal place.**

3. Unidentified abnormal symbols appear in Lines 34, 35, and 37. Please remove all garbled characters and standardize the text.

**RESPONSE: These are formatting artifacts. We reviewed the manuscript and found that several non-standard hyphen characters were present in the text and appear to have rendered incorrectly on translation to PDF. These have been replaced with standard hyphens, and the manuscript has been checked for similar garbled or non-standard characters throughout. We also standardized terminology and formatting across the affected text. If remaining abnormal symbols are left in the manuscript after conversion this may be something we need to sort with the copy editor.**

4. The statements in Lines 76-78 require supporting literature. Please add appropriate references to validate the claims.

**RESPONSE: We have revised this section and added more directly relevant supporting literature. A more fulsome description of the intervention framework is provided in the Methods section under Participants.**

5. The content in Lines 78-79 is grammatically incomplete. Please revise it into a complete sentence.

**RESPONSE: Thank you for identifying this grammatical issue. We have revised the sentence into a complete grammatical structure and clarified the description of commonly observed recovery trajectory patterns.**

“*Across studies, commonly observed patterns include a favorable or rapid recovery trajectory and a persistent or minimal recovery trajectory, with one or more intermediate, delayed, or fluctuating patterns identified in many cohorts*”

6. Supplement relevant references for the statements in Lines 99-100 to strengthen the theoretical basis.

**RESPONSE: Thank you for this comment. The baseline phenotypes referenced in this section are described in a companion paper that is currently under peer review and therefore cannot yet be cited as a published or accepted source. For now we have used the placeholder [cite when available] to flag this, and will have to add the relevant citation when it becomes available. That companion paper has already been revised and resubmitted and we anticipate a rapid turnaround on it.**

7. Lines 113-117 only present research objectives. To improve the theoretical depth of the study, explicit research hypotheses should be added accordingly.

**RESPONSE: We have added explicit hypotheses to the end of the Introduction section**

8. The paragraph break at Line 152 is unnecessary. Please merge the paragraph with the previous text.

**RESPONSE: Done as suggested.**

9. The manuscript contains five tables but only two figures, leading to unbalanced visual presentation. It is recommended to convert two to three tables into figures for better result visualization.

**RESPONSE:** **To improve the balance between tabular and graphical presentation and make the pattern of results easier to interpret, we have added some colour to Table 4 with a heatmap-style figure. The new figure presents the same outcome proportions across the baseline LPA classes using a common 0–100% scale, allowing the overall pattern of recovery trajectories and point outcomes to be appreciated more readily while preserving the underlying percentages. The corresponding text and figure numbering have been updated accordingly.**

10. The formatting of Tables 1-3 is inconsistent with that of Tables 4-5. Please unify table styles throughout the manuscript.

**RESPONSE: All tables have been formatted consistently.**

11. The “work type” variable in Table 1 is completely blank. Please either supplement the missing data or remove this variable from the table.

**RESPONSE: Thank you, this was an oversight. The “Work type” row was an inadvertently retained placeholder and was not used in the analyses reported in this manuscript. We have therefore removed this row from Table 1.**

12. Abnormal symbols exist in Line 423. Please check and correct the text.

**RESPONSE: We have rechecked the affected text and replaced non-standard character encoding in this section.**

13. The parenthetical expression “(approximately)” in Line 429 is redundant and should be removed.

**RESPONSE: Removed as suggested.**

14. The conclusion section starting at Line 502 is excessively verbose. Please condense and refine it to highlight core findings and implications.

**RESPONSE: We have substantially condensed this section to emphasize the principal findings, the statistical imprecision associated with the smallest phenotype, and the need for prospective validation of the longer-term prognostic and clinical utility of this approach.**

15. Obvious tracked revision marks remain in Lines 522-524. Please clean up all editing traces in the final version.

**RESPONSE: All tracked changes, comments, and residual editing marks have been removed from the clean, revised manuscript.**

16. To improve data transparency and reproducibility, the authors are encouraged to upload the raw dataset to a public repository such as Figshare and add a corresponding data availability statement.

**RESPONSE: We had previously uploaded the open dataset to our institutions approved data repository (Borealis) however the Editor has flagged that it is not one of the approved repositories for PLoS One. So we have uploaded it to the OSF site where this protocol was originally registered, which does appear on the list of PLoS One approved registries.**

17. Please double-check the reference format and confirm whether the prefix “p.” is required before page numbers for consistent journal formatting.

**RESPONSE: We have reformatted the reference list accordingly and checked all references for consistency with PLOS ONE style.**

18. Please revise the horizontal axis titles of Figure 1 and Figure 2 uniformly to “Time”.

**RESPONSE: Revised as suggested.**

Reviewer #3: General Comments
The authors address a relevant and generally well-justified topic. The manuscript evaluates a considerable number of outcomes, which provides a comprehensive perspective on the research question. However, the large amount of information also increases the complexity and length of the manuscript, making some methodological aspects and results difficult to follow at times. Nevertheless, I believe the manuscript has good potential. The comments below are intended to help improve the clarity, organization, and interpretation of the information presented.

Introduction
*1. The authors provide a clear description of the problem addressed in the Introduction, particularly regarding the variability in individual responses during the rehabilitation process. However, limited information is provided regarding the rehabilitation programs currently recommended or commonly implemented in this context. Although I understand that this is not the primary focus of the manuscript, a brief description of the rehabilitation approaches associated with these heterogeneous responses could provide useful context for readers and strengthen the rationale for the study.*

**RESPONSE: We have briefly introduced the Ontario WSIB Musculoskeletal Program of Care received by participants in the present cohort within the Introduction (para 2), noting that it provides a standardized eight-week framework while allowing treatment selection, frequency, and intensity to be individualized according to clinical and functional needs. More detailed information regarding the structure and components of the program is provided in the Methods to avoid unnecessary duplication.**

Methods
*1. The manuscript would benefit from a more detailed description of the rehabilitation program implemented in the study. Although the authors mention the main practices included in the intervention, additional information regarding its structure and implementation would improve reproducibility. I recognize that the primary focus of the study is to understand the variability in responses; nevertheless, readers should have sufficient information about the rehabilitation program from which these responses were derived. I therefore encourage the authors to provide additional details that would allow the intervention to be adequately understood and potentially replicated.*

**RESPONSE: We have expanded the Methods to provide a more detailed description of the Ontario WSIB Musculoskeletal Program of Care.**

2. Some methodological aspects are described only briefly, with the authors referring readers to a previous study that has not yet been published for additional details. This may limit the reader's ability to fully evaluate the methodology of the present study, as the referenced information is currently unavailable. I encourage the authors to consider providing the essential methodological information directly in the present manuscript. Alternatively, references to the unpublished manuscript could be reconsidered when they serve as the primary source for methodological details necessary to understand or reproduce the study.

**RESPONSE: We acknowledge the lack of clarity this imposes on the reader, though remain committed to ensuring both manuscripts are published for public access, appraisal, and to support ongoing work in the field. We had submitted the prior manuscript to this one, in which the baseline phenotypic profiles are derived, several months prior though a delay in the review process has caused a bit of a bottleneck. That prior development paper has now been revised and resubmitted and is currently under second review at PLoS One. As it too describes complex analyses we are trying to avoid any sense of duplication or, worse, self-plagiarism between the two. For now our best attempt has been to flag where the companion paper needs to be cited [cite when available] so it is easy to add when ready. We are optimistic that prior paper will receive a quick turnaround and will be published before this one.**

3. The manuscript could benefit from the inclusion of a figure illustrating the study design and assessment timeline. In particular, the measures could be presented in a schematic figure indicating the time points at which the “trajectory measures” and “point outcomes” were obtained. Such a figure could facilitate understanding of the study procedures and provide readers with a clearer overview of when each assessment was performed.

**RESPONSE: Done as requested (adding a follow diagram)**

*4. Regarding sample size, I suggest that the authors explicitly describe the sampling strategy used to recruit participants (e.g., non-probability or convenience sampling). Although it appears clear that no formal sample size calculation was performed, the sampling approach itself is not sufficiently described. Providing this information would improve methodological transparency.*

**RESPONSE: This is an example of a methodological aspect that has been described in detail in the companion paper, though the reviewer is quite right it should have been better detailed here. We have revised the Participants section to clarify that participants were recruited using a convenience sampling approach. We have also clarified in the Sample Size section that no formal a priori sample-size calculation was performed. The existing text in this section explains the considerations underlying the recruitment target for growth mixture modeling and reports the final sample available for each trajectory analysis. We have attempted to make clear here and throughout the paper that this is exploratory, discovery-based work that will require further validation.**

Results
*1. A similar consideration applies to the initial part of the Results section, particularly the subsection entitled “Fidelity and Data Cleaning.” The inclusion of a participant flowchart could provide a clearer and more intuitive representation of participant progression throughout the study, including losses to follow-up and the corresponding reasons. I recognize that journal restrictions regarding the number of figures and tables may influence how this information can be presented. Therefore, the authors may consider this suggestion according to the journal's formatting requirements and available space.*

*2. I suggest reconsidering the placement of the information regarding “Point Outcomes,” which is currently presented in the Statistical Analysis section. This information appears to describe how the outcomes were defined or assessed and, therefore, may be more appropriately included in the Measures section. This change could improve the organization and logical flow of the Methods section.*

**RESPONSE: Thank you for this thoughtful suggestion. We agree that information describing the point outcomes themselves belongs in the Measures section. In the current manuscript, the Measures section describes the three point outcomes, the timing of their assessment, their response categories, and the source of each outcome. The “Point Outcomes” subsection within the Statistical Analysis section is intended specifically to describe the analytical recoding of these variables into binary “Recovered” and “Not Recovered” categories for the subsequent analyses. We have therefore retained the current organization to distinguish outcome measurement from analytical processing. We believe this separation provides a clear distinction between how the outcomes were collected and how they were subsequently operationalized for analysis.**

*3. Figures 1 and 2 would benefit from more detailed legends to facilitate interpretation without requiring readers to repeatedly consult the main text. In addition, I encourage the authors to reconsider whether presenting the same findings in both figures (Figures 1 and 2) and a table (Table 2) is necessary. If these elements present essentially the same results, selecting the format that communicates the findings most effectively could reduce redundancy and improve the overall conciseness of the manuscript.*

**RESPONSE: We carefully considered the potential redundancy between the trajectory figures and corresponding tables. We elected to retain both because they serve complementary purposes: the figures provide a clear visual representation of the recovery patterns, whereas Tables 2 and 3 provide the exact numerical estimates and statistical comparisons. We also retained the current legends to avoid duplicating information already provided immediately adjacent to the figures in the Results section. However, and per a comment from another reviewer, we have replaced Table 4 with a figure instead, hoping to strike a better balance between text and images in what is indeed a dense manuscript.**

*4. The classifications “Rapid Recovery,” “Incomplete Recovery,” and “No Recovery” appear to be introduced primarily in the Results section. For methodological clarity, I suggest describing the classification approach and the criteria used to define these categories in the Methods section before presenting the corresponding results. This would allow readers to understand how participants or outcomes were classified before interpreting the findings.*

**RESPONSE: We have clarified the methods to indicate that the trajectory classes were empirically derived through GMM rather than defined using *a priori* clinical thresholds. We now specify that participants were assigned to classes based on their highest posterior probability of membership and that the descriptive labels (e.g., Rapid Recovery, Incomplete Recovery, and No Recovery) were assigned post hoc based on the estimated level and pattern of change over time and did not influence class assignment (lines 245 to 250).**

*5. Several abbreviations are used throughout the tables. I recommend defining all abbreviations in the corresponding table legends or footnotes so that each table can be interpreted independently from the main text.*

**RESPONSE: We have reviewed all tables and added definitions for the abbreviations used in each table to the corresponding footnotes. Each table can therefore now be interpreted independently without requiring readers to refer back to the main text.**

Discussion
1. At the beginning of the Discussion, findings from previous studies (e.g., “Study One”) are presented in close proximity to the findings of the current study. In some instances, this may make it difficult for readers to distinguish between results derived from the present analysis and those reported previously. I suggest emphasizing the main findings of the current study at the beginning of the Discussion and subsequently using findings from previous studies to contextualize, compare, and interpret the present results. This consideration may also be relevant to other parts of the Discussion. Maintaining the findings of the current manuscript as the central focus throughout the section could improve clarity and strengthen the interpretation of the study.

**RESPONSE: On another reading with fresh eyes we can see what the reviewer is seeing. While this manuscript only makes sense in the context of the two companions, the flow of this opening paragraph did run the risk of conflation across analyses. We’ve restructured it to open with the focus of the current analysis, then allude to the two companions, then close this paragraph with the overall view that we have now closed ‘the loop’ on this set of analyses. We hope this flows better to the reviewer, as it does now to us.**

Conclusion
1. The Conclusion could be more concise and focused, as some statements appear to reiterate results already described in previous sections of the manuscript. I encourage the authors to provide a broader synthesis of the main findings rather than restating individual results. The Conclusion could emphasize the most relevant overall findings and their implications. In addition, I suggest framing the conclusions more explicitly in relation to the specific rehabilitation program evaluated in this study, as the observed findings and response patterns should be interpreted within the context of this particular rehabilitation model.

**RESPONSE: We have substantially revised the Conclusion section, noted as overly verbose by two reviewers. In doing so we have reduced its length by 50%, from 196 words to 96 words and agree that it reads much more concisely and clearly for having done so.**
