# Supplementary material for: Defining and predicting outcomes of a work-injuries rehabilitation program through multivariate baseline phenotypes and longitudinal modeling
Source: PLoS One. 2026 Nov 4;21(11):e0362083. doi: 10.1371/journal.pone.0362083 (PMC13734445; doi:10.1371/journal.pone.0362083)
Supplement: Peer review history

# Peer review: Decision Letter 0

**DOI:** 10.1371/journal.pone.0362083.r001

**Authors:** Tomasz W. Kaminski

3 Aug 2026

Dear Dr. Walton,

Thank you for submitting your manuscript to PLOS ONE. After careful consideration, we feel that it has merit but does not fully meet PLOS ONE’s publication criteria as it currently stands. Therefore, we invite you to submit a revised version of the manuscript that addresses the points raised during the review process.

Please submit your revised manuscript by Oct 02 2026 11:59PM. If you will need more time than this to complete your revisions, please reply to this message or contact the journal office at plosone@plos.org. When you're ready to submit your revision, log on to https://www.editorialmanager.com/pone/ and select the 'Submissions Needing Revision' folder to locate your manuscript file.

- A letter that responds to each point raised by the academic editor and reviewer(s). You should upload this letter as a separate file labeled 'Response to Reviewers'.
- A marked-up copy of your manuscript that highlights changes made to the original version. You should upload this as a separate file labeled 'Revised Manuscript with Track Changes'.
- An unmarked version of your revised paper without tracked changes. You should upload this as a separate file labeled 'Manuscript'.

If you would like to make changes to your financial disclosure, please include your updated statement in your cover letter. Guidelines for resubmitting your figure files are available below the reviewer comments at the end of this letter.

If applicable, we recommend that you deposit your laboratory protocols in protocols.io to enhance the reproducibility of your results. Protocols.io assigns your protocol its own identifier (DOI) so that it can be cited independently in the future. For instructions see: https://journals.plos.org/plosone/s/submission-guidelines#loc-laboratory-protocols. Additionally, PLOS ONE offers an option for publishing peer-reviewed Lab Protocol articles, which describe protocols hosted on protocols.io. Read more information on sharing protocols at https://plos.org/protocols?utm_medium=editorial-email&utm_source=authorletters&utm_campaign=protocols.

As the corresponding author, your ORCID iD is verified in the submission system and will appear in the published article. PLOS supports the use of ORCID, and we encourage all coauthors to register for an ORCID iD and use it as well. Please encourage your coauthors to verify their ORCID iD within the submission system before final acceptance, as unverified ORCID iDs will not appear in the published article. *Only*  the individual author can complete the verification step; PLOS staff *cannot*  verify ORCID iDs on behalf of authors.

We look forward to receiving your revised manuscript.

Kind regards,

Tomasz W. Kaminski

Academic Editor

PLOS One

Journal Requirements:

When submitting your revision, we need you to address these additional requirements.

1.Please ensure that your manuscript meets PLOS ONE's style requirements, including those for file naming. The PLOS ONE style templates can be found at

https://journals.plos.org/plosone/s/file?id=wjVg/PLOSOne_formatting_sample_main_body.pdf and

https://journals.plos.org/plosone/s/file?id=ba62/PLOSOne_formatting_sample_title_authors_affiliations.pdf

2. Please note that PLOS One has specific guidelines on code sharing for submissions in which author-generated code underpins the findings in the manuscript. In these cases, all author-generated code must be made available without restrictions upon publication of the work. Please review our guidelines at https://journals.plos.org/plosone/s/materials-and-software-sharing#loc-sharing-code and ensure that your code is shared in a way that follows best practice and facilitates reproducibility and reuse.

3. We note that the grant information you provided in the ‘Funding Information’ and ‘Financial Disclosure’ sections do not match.

When you resubmit, please ensure that you provide the correct grant numbers for the awards you received for your study in the ‘Funding Information’ section.

4. Thank you for stating the following financial disclosure:

“Ontario Workplace Safety and Insurance Board Research Grant awarded to D. Walton, held and managed by the Western University Research Finance Office.”

Please state what role the funders took in the study.  If the funders had no role, please state: "The funders had no role in study design, data collection and analysis, decision to publish, or preparation of the manuscript."

If this statement is not correct you must amend it as needed.

Please include this amended Role of Funder statement in your cover letter; we will change the online submission form on your behalf.

5. Thank you for stating the following in the Competing Interests section:

“I have read the journal's policy and the authors of this manuscript have the following competing interests: D. Walton is co-author of a text book published in 2020 (pub: Singing Dragon Press) in which the seven-axis framework from which the baseline variables used in this study was derived. The results of this study have no influence on the contents of that book.”

We note that one or more of the authors are employed by a commercial company: “CBI Health Group Inc., Ontario, Canada and Singing Dragon Press”

1. Please provide an amended Funding Statement declaring this commercial affiliation, as well as a statement regarding the Role of Funders in your study. If the funding organization did not play a role in the study design, data collection and analysis, decision to publish, or preparation of the manuscript and only provided financial support in the form of authors' salaries and/or research materials, please review your statements relating to the author contributions, and ensure you have specifically and accurately indicated the role(s) that these authors had in your study. You can update author roles in the Author Contributions section of the online submission form.

Please also include the following statement within your amended Funding Statement.

“The funder provided support in the form of salaries for authors “DW and TC”, but did not have any additional role in the study design, data collection and analysis, decision to publish, or preparation of the manuscript. The specific roles of these authors are articulated in the ‘author contributions’ section.”

If your commercial affiliation did play a role in your study, please state and explain this role within your updated Funding Statement.

2. Please also provide an updated Competing Interests Statement declaring this commercial affiliation along with any other relevant declarations relating to employment, consultancy, patents, products in development, or marketed products, etc.

Within your Competing Interests Statement, please confirm that this commercial affiliation does not alter your adherence to all PLOS ONE policies on sharing data and materials by including the following statement: "This does not alter our adherence to  PLOS ONE policies on sharing data and materials.” (as detailed online in our guide for authors http://journals.plos.org/plosone/s/competing-interests) . If this adherence statement is not accurate and  there are restrictions on sharing of data and/or materials, please state these. Please note that we cannot proceed with consideration of your article until this information has been declared.

Please include both an updated Funding Statement and Competing Interests Statement in your cover letter. We will change the online submission form on your behalf.

6. Thank you for stating the following in the Acknowledgments Section of your manuscript:

“We thank the staff and clients at CBI Health Inc. in Ontario for their contributions to the 522 success of this study. We also thank Lynn Cooper from the Canadian Injured Workers 523 Alliance for her early input in shaping the study concept and helping ensure its 524 relevance to the lived experiences and rehabilitation priorities of injured workers.”

We note that you have provided funding information that is not currently declared in your Funding Statement. However, funding information should not appear in the Acknowledgments section or other areas of your manuscript. We will only publish funding information present in the Funding Statement section of the online submission form.

Please remove any funding-related text from the manuscript and let us know how you would like to update your Funding Statement. Currently, your Funding Statement reads as follows:

“Ontario Workplace Safety and Insurance Board Research Grant awarded to D. Walton, held and managed by the Western University Research Finance Office.”

Please include your amended statements within your cover letter; we will change the online submission form on your behalf.

7. Thank you for uploading your study's underlying data set. Unfortunately, the repository you have noted in your Data Availability statement does not qualify as an acceptable data repository according to PLOS's standards.

At this time, please upload the minimal data set necessary to replicate your study's findings to a stable, public repository (such as figshare or Dryad) and provide us with the relevant URLs, DOIs, or accession numbers that may be used to access these data. For a list of recommended repositories and additional information on PLOS standards for data deposition, please see https://journals.plos.org/plosone/s/recommended-repositories.

8. Please include your full ethics statement in the ‘Methods’ section of your manuscript file. In your statement, please include the full name of the IRB or ethics committee who approved or waived your study, as well as whether or not you obtained informed written or verbal consent. If consent was waived for your study, please include this information in your statement as well.

9. We notice that your supplementary figures are uploaded with the file type 'Figure'. Please amend the file type to 'Supporting Information'. Please ensure that each Supporting Information file has a legend listed in the manuscript after the references list.

10. If the reviewer comments include a recommendation to cite specific previously published works, please review and evaluate these publications to determine whether they are relevant and should be cited. There is no requirement to cite these works unless the editor has indicated otherwise.

Additional Editor Comments:

Dear Authors,

Thank you for submitting your manuscript. The reviewers agree that the study addresses an important and clinically relevant topic and has the potential to contribute to the literature on risk-stratified rehabilitation.

However, substantial revisions are required before the manuscript can be considered further. The main concerns relate to the stability and interpretation of the statistical models, the relatively small sample size, attrition and missing-data bias, limited description of the rehabilitation intervention, incomplete methodological details, and the organization and clarity of the results and discussion.

Please revise the manuscript carefully in response to all reviewer comments and provide a detailed point-by-point response describing the changes made.

Sincerely,

Tomasz W Kaminski

The Academic Editor

[Note: HTML markup is below. Please do not edit.]

Reviewer's Responses to Questions

**Comments to the Author**

1. Is the manuscript technically sound, and do the data support the conclusions?

Reviewer #1: Yes

Reviewer #2: Yes

Reviewer #3: Partly

**********

2. Has the statistical analysis been performed appropriately and rigorously?

Reviewer #1: Yes

Reviewer #2: Yes

Reviewer #3: Yes

**********

3. Have the authors made all data underlying the findings in their manuscript fully available?

The PLOS Data policy

Reviewer #1: Yes

Reviewer #2: No

Reviewer #3: Yes

**********

4. Is the manuscript presented in an intelligible fashion and written in standard English?

Reviewer #1: Yes

Reviewer #2: Yes

Reviewer #3: Yes

**********

Reviewer #1: Dear Authors,

As a reviewer for this manuscript, I provide below my considerations and suggestions for improvement to strengthen the article "Defining and predicting outcomes of a work-injuries rehabilitation program through multivariate baseline phenotypes and longitudinal modeling" for potential publication in PLOS One. The study addresses a highly relevant clinical topic—the phenotyping of patients to predict recovery trajectories in injured workers—and employs robust, advanced statistical techniques.

The following points warrant attention:

1. Statistical Robustness and Sample Size

While the use of Growth Mixture Modeling (GMM) and Latent Profile Analysis (LPA) is a strength of this work, the sample size (N = 145) is on the lower end of the recommended range for these techniques, which typically require between 100 to 300 participants to ensure class stability.

• Suggestion: I recommend expanding the discussion on how the sample size may have influenced model convergence, particularly for pain (NPRS), where 2- and 4-class models were found to be inadmissible.

2. Confidence Intervals and Stability of the "Complex/Central" Group

The results indicate an extremely high Odds Ratio (OR) of 150.00 for the "Complex/Central" group in predicting "No Improvement" trajectories in quality of life, but with a very wide 95% Confidence Interval (18.53–1214.16).

• Suggestion: It is imperative to emphasize in the discussion that these values are effect estimates and may be unstable due to the small number of participants in this specific phenotype (representing only 8.5% of the sample). Please avoid definitive conclusions based on such high point estimates without clearly noting the statistical imprecision.

3. Constraints in the Quality of Life Model (EQ-5D-5L)

You mentioned that to achieve model convergence for the quality of life trajectories, it was necessary to constrain residual variances to be equal over time within each class.

• Suggestion: Provide a more detailed explanation in the text (beyond the limitations section) regarding how this constraint might have oversimplified the class structure. Specifically, discuss if it potentially masked individual heterogeneity during the initial phase of rehabilitation.

4. Updating Missing Citations

The manuscript contains several "[cite when available]" placeholders in critical sections, such as the description of input variables for the LPA and the grounding of the screening algorithms.

• Suggestion: Before final submission, these placeholders must be replaced with full references (or indicated as "in press" where applicable). Clarity regarding the "seven-axis framework" is essential for the replicability of the study.

5. Attrition Rate

The retention rate was approximately 77%, which is slightly below the commonly accepted 80% standard for high-quality prognostic studies.

• Suggestion: Briefly provide a comparative analysis between participants who completed the study and those lost to follow-up (regarding demographic characteristics and baseline pain) to rule out potential systematic selection bias.

6. Detailing the Intervention

The article focuses on prediction but describes the intervention somewhat generically as a "standardized program of care".

• Suggestion: While the treatment is guided by WSIB protocols, adding a more detailed description of the average session frequency (e.g., 1–2 times per week) and specific components (exercise, manual therapy, education) would help the reader understand the context of what the "No Recovery" groups experienced.

7. Follow-up Period

The study evaluates outcomes up to 8 weeks, which is a short-term window in the context of occupational injuries.

• Suggestion: Discuss whether initial phenotyping might also hold value for predicting long-term outcomes (e.g., 6 or 12 months), as tools like STarT Back are often validated over longer timeframes.

In conclusion, this study is innovative in demonstrating that phenotypes derived entirely from brief self-reports (taking about 5 minutes) can effectively stratify risk in acutely injured workers. With these adjustments, the work will make a significant impact on the literature regarding risk-stratified musculoskeletal rehabilitation.

Reviewer #2: Overall, this study investigated whether multidimensional baseline phenotypes based on brief self-reported data can predict early pain and HRQoL trajectories and short-term rehabilitation outcomes among injured workers. The results demonstrate that baseline multivariate phenotyping effectively stratifies patients with distinct rehabilitation prognoses. This is a well-structured and interesting manuscript. Several minor and moderate revisions are suggested to improve the presentation, rigor, and readability of this work.

1. The sentence in Lines 25-28 is overly lengthy and difficult to follow. Please rephrase and simplify it for better readability.

2. The approximate proportion values (≈55%, 20%, 15%, 10%) in Lines 32-33 are inappropriate for formal reporting. Please provide exact percentages with one decimal place to ensure consistent formatting.

3. Unidentified abnormal symbols appear in Lines 34, 35, and 37. Please remove all garbled characters and standardize the text.

4. The statements in Lines 76-78 require supporting literature. Please add appropriate references to validate the claims.

5. The content in Lines 78-79 is grammatically incomplete. Please revise it into a complete sentence.

6. Supplement relevant references for the statements in Lines 99-100 to strengthen the theoretical basis.

7. Lines 113-117 only present research objectives. To improve the theoretical depth of the study, explicit research hypotheses should be added accordingly.

8. The paragraph break at Line 152 is unnecessary. Please merge the paragraph with the previous text.

9. The manuscript contains five tables but only two figures, leading to unbalanced visual presentation. It is recommended to convert two to three tables into figures for better result visualization.

10. The formatting of Tables 1-3 is inconsistent with that of Tables 4-5. Please unify table styles throughout the manuscript.

11. The “work type” variable in Table 1 is completely blank. Please either supplement the missing data or remove this variable from the table.

12. Abnormal symbols exist in Line 423. Please check and correct the text.

13. The parenthetical expression “(approximately)” in Line 429 is redundant and should be removed.

14. The conclusion section starting at Line 502 is excessively verbose. Please condense and refine it to highlight core findings and implications.

15. Obvious tracked revision marks remain in Lines 522-524. Please clean up all editing traces in the final version.

16. To improve data transparency and reproducibility, the authors are encouraged to upload the raw dataset to a public repository such as Figshare and add a corresponding data availability statement.

17. Please double-check the reference format and confirm whether the prefix “p.” is required before page numbers for consistent journal formatting.

18. Please revise the horizontal axis titles of Figure 1 and Figure 2 uniformly to “Time”.

Reviewer #3: General Comments

The authors address a relevant and generally well-justified topic. The manuscript evaluates a considerable number of outcomes, which provides a comprehensive perspective on the research question. However, the large amount of information also increases the complexity and length of the manuscript, making some methodological aspects and results difficult to follow at times. Nevertheless, I believe the manuscript has good potential. The comments below are intended to help improve the clarity, organization, and interpretation of the information presented.

Introduction

1. The authors provide a clear description of the problem addressed in the Introduction, particularly regarding the variability in individual responses during the rehabilitation process. However, limited information is provided regarding the rehabilitation programs currently recommended or commonly implemented in this context. Although I understand that this is not the primary focus of the manuscript, a brief description of the rehabilitation approaches associated with these heterogeneous responses could provide useful context for readers and strengthen the rationale for the study.

Methods

1. The manuscript would benefit from a more detailed description of the rehabilitation program implemented in the study. Although the authors mention the main practices included in the intervention, additional information regarding its structure and implementation would improve reproducibility. I recognize that the primary focus of the study is to understand the variability in responses; nevertheless, readers should have sufficient information about the rehabilitation program from which these responses were derived. I therefore encourage the authors to provide additional details that would allow the intervention to be adequately understood and potentially replicated.

2. Some methodological aspects are described only briefly, with the authors referring readers to a previous study that has not yet been published for additional details. This may limit the reader's ability to fully evaluate the methodology of the present study, as the referenced information is currently unavailable. I encourage the authors to consider providing the essential methodological information directly in the present manuscript. Alternatively, references to the unpublished manuscript could be reconsidered when they serve as the primary source for methodological details necessary to understand or reproduce the study.

3. The manuscript could benefit from the inclusion of a figure illustrating the study design and assessment timeline. In particular, the measures could be presented in a schematic figure indicating the time points at which the “trajectory measures” and “point outcomes” were obtained. Such a figure could facilitate understanding of the study procedures and provide readers with a clearer overview of when each assessment was performed.

4. Regarding sample size, I suggest that the authors explicitly describe the sampling strategy used to recruit participants (e.g., non-probability or convenience sampling). Although it appears clear that no formal sample size calculation was performed, the sampling approach itself is not sufficiently described. Providing this information would improve methodological transparency.

Results

1. A similar consideration applies to the initial part of the Results section, particularly the subsection entitled “Fidelity and Data Cleaning.” The inclusion of a participant flowchart could provide a clearer and more intuitive representation of participant progression throughout the study, including losses to follow-up and the corresponding reasons. I recognize that journal restrictions regarding the number of figures and tables may influence how this information can be presented. Therefore, the authors may consider this suggestion according to the journal's formatting requirements and available space.

2. I suggest reconsidering the placement of the information regarding “Point Outcomes,” which is currently presented in the Statistical Analysis section. This information appears to describe how the outcomes were defined or assessed and, therefore, may be more appropriately included in the Measures section. This change could improve the organization and logical flow of the Methods section.

3. Figures 1 and 2 would benefit from more detailed legends to facilitate interpretation without requiring readers to repeatedly consult the main text. In addition, I encourage the authors to reconsider whether presenting the same findings in both figures (Figures 1 and 2) and a table (Table 2) is necessary. If these elements present essentially the same results, selecting the format that communicates the findings most effectively could reduce redundancy and improve the overall conciseness of the manuscript.

4. The classifications “Rapid Recovery,” “Incomplete Recovery,” and “No Recovery” appear to be introduced primarily in the Results section. For methodological clarity, I suggest describing the classification approach and the criteria used to define these categories in the Methods section before presenting the corresponding results. This would allow readers to understand how participants or outcomes were classified before interpreting the findings.

5. Several abbreviations are used throughout the tables. I recommend defining all abbreviations in the corresponding table legends or footnotes so that each table can be interpreted independently from the main text.

Discussion

1. At the beginning of the Discussion, findings from previous studies (e.g., “Study One”) are presented in close proximity to the findings of the current study. In some instances, this may make it difficult for readers to distinguish between results derived from the present analysis and those reported previously. I suggest emphasizing the main findings of the current study at the beginning of the Discussion and subsequently using findings from previous studies to contextualize, compare, and interpret the present results. This consideration may also be relevant to other parts of the Discussion. Maintaining the findings of the current manuscript as the central focus throughout the section could improve clarity and strengthen the interpretation of the study.

Conclusion

1. The Conclusion could be more concise and focused, as some statements appear to reiterate results already described in previous sections of the manuscript. I encourage the authors to provide a broader synthesis of the main findings rather than restating individual results. The Conclusion could emphasize the most relevant overall findings and their implications. In addition, I suggest framing the conclusions more explicitly in relation to the specific rehabilitation program evaluated in this study, as the observed findings and response patterns should be interpreted within the context of this particular rehabilitation model.

**********

what does this mean?). If published, this will include your full peer review and any attached files.

If you choose “no”, your identity will remain anonymous but your review may still be made public.

**Do you want your identity to be public for this peer review?**  For information about this choice, including consent withdrawal, please see our Privacy Policy

Reviewer #1: No

Reviewer #2: No

Reviewer #3: No

**********

[NOTE: If reviewer comments were submitted as an attachment file, they will be attached to this email and accessible via the submission site. Please log into your account, locate the manuscript record, and check for the action link "View Attachments". If this link does not appear, there are no attachment files.]

To ensure your figures meet our technical requirements, please review our figure guidelines: https://journals.plos.org/plosone/s/figures

You may also use PLOS’s free figure tool, NAAS, to help you prepare publication quality figures: https://journals.plos.org/plosone/s/figures#loc-tools-for-figure-preparation.

NAAS will assess whether your figures meet our technical requirements by comparing each figure against our figure specifications.

Attachment Submitted filename: Reviewer_PO.docx

# Author comment: Author response to Decision Letter 1

**DOI:** 10.1371/journal.pone.0362083.r002

6 Oct 2026

Our point-by-point response to the reviewer comments are included in the response to reviewers document uploaded with the rest of the submission.

Attachment Submitted filename: Response to Reviewers.docx

# Peer review: Decision Letter 1

**DOI:** 10.1371/journal.pone.0362083.r003

**Authors:** Tomasz W. Kaminski

20 Oct 2026

Defining and predicting outcomes of a work-injuries rehabilitation program through multivariate baseline phenotypes and longitudinal modeling

PONE-D-26-29545R1

Dear Dr. Walton,

We’re pleased to inform you that your manuscript has been judged scientifically suitable for publication and will be formally accepted for publication once it meets all outstanding technical requirements.

Within one week, you’ll receive an e-mail detailing the required amendments. When these have been addressed, you’ll receive a formal acceptance letter and your manuscript will be scheduled for publication.

An invoice will be generated when your article is formally accepted. Please note, if your institution has a publishing partnership with PLOS and your article meets the relevant criteria, all or part of your publication costs will be covered. Please make sure your user information is up-to-date by logging into Editorial Manager at Editorial Manager® and clicking the ‘Update My Information' link at the top of the page. For questions related to billing, please contact billing support.

If your institution or institutions have a press office, please notify them about your upcoming paper to help maximize its impact. If they’ll be preparing press materials, please inform our press team as soon as possible -- no later than 48 hours after receiving the formal acceptance. Your manuscript will remain under strict press embargo until 2 pm Eastern Time on the date of publication. For more information, please contact onepress@plos.org.

Kind regards,

Tomasz W. Kaminski

Academic Editor

PLOS One

Reviewers' comments:

Reviewer's Responses to Questions

**Comments to the Author**

Reviewer #1: All comments have been addressed

Reviewer #2: All comments have been addressed

**********

2. Is the manuscript technically sound, and do the data support the conclusions?

Reviewer #1: Yes

Reviewer #2: Yes

**********

3. Has the statistical analysis been performed appropriately and rigorously?

Reviewer #1: Yes

Reviewer #2: Yes

**********

4. Have the authors made all data underlying the findings in their manuscript fully available?

The PLOS Data policy

Reviewer #1: Yes

Reviewer #2: Yes

**********

5. Is the manuscript presented in an intelligible fashion and written in standard English?

Reviewer #1: Yes

Reviewer #2: Yes

**********

Reviewer #1: (No Response)

Reviewer #2: The author have addressed all previous comments. I am happy with these revisions, no further comments.

**********

what does this mean?). If published, this will include your full peer review and any attached files.

If you choose “no”, your identity will remain anonymous but your review may still be made public.

**Do you want your identity to be public for this peer review?**  For information about this choice, including consent withdrawal, please see our Privacy Policy

Reviewer #1: No

Reviewer #2: No

**********

# Editor report: Acceptance letter

**DOI:** 10.1371/journal.pone.0362083.r004

**Authors:** Tomasz W. Kaminski

PONE-D-26-29545R1

PLOS One

Dear Dr. Walton,

I'm pleased to inform you that your manuscript has been deemed suitable for publication in PLOS One. Congratulations! Your manuscript is now being handed over to our production team.

At this stage, our production department will prepare your paper for publication. This includes ensuring the following:

* All references, tables, and figures are properly cited

* All relevant supporting information is included in the manuscript submission,

* There are no issues that prevent the paper from being properly typeset

You will receive further instructions from the production team, including instructions on how to review your proof when it is ready. Please keep in mind that we are working through a large volume of accepted articles, so please give us a few days to review your paper and let you know the next and final steps.

Lastly, if your institution or institutions have a press office, please let them know about your upcoming paper now to help maximize its impact. If they'll be preparing press materials, please inform our press team within the next 48 hours. Your manuscript will remain under strict press embargo until 2 pm Eastern Time on the date of publication. For more information, please contact onepress@plos.org.

You will receive an invoice from PLOS for your publication fee after your manuscript has reached the completed accept phase. If you receive an email requesting payment before acceptance or for any other service, this may be a phishing scheme. Learn how to identify phishing emails and protect your accounts at https://explore.plos.org/phishing.

If we can help with anything else, please email us at customercare@plos.org.

Thank you for submitting your work to PLOS One and supporting open access.

Kind regards,

PLOS One Editorial Office Staff

on behalf of

Dr. Tomasz W. Kaminski

Academic Editor

PLOS One
